# A unified MAP–EM approach to stable Gaussian mixture clustering with priors, graphs, and split–merge adaptation for document clustering

**DOI:** 10.3389/frai.2026.1816534

**Published:** 2026-08-24

**Authors:** Sumathi Subbarayan, G. Hannah Grace

**Affiliations:** Department of Mathematics, School of Advanced Sciences, Vellore Institute of Technology Chennai, Chennai, Tamil Nadu, India

**Keywords:** convergence analysis, document clustering, EM algorithm, Gaussian mixture model, graph Laplacian, model-based clustering, normal–inverse–Wishart (NIW) prior, split–merge

## Abstract

**Introduction:**

Clustering high-dimensional and noisy data remains challenging for conventional expectation–maximization (EM) methods as overlapping clusters, sparse features, and outliers can lead to covariance degeneracy and unstable parameter estimates. This research aims to improve clustering performance in high-dimensional, noisy settings by developing a robust maximum a posteriori expectation–maximization (MAP–EM) framework that integrates prior regularization, geometric structure, and outlier handling. Traditional EM-based clustering methods often struggle in the presence of overlapping clusters, high-dimensional features, and outliers, leading to degenerate covariance and unstable parameter estimates.

**Methods:**

The proposed MAP–EM model improves reliability by combining normal—inverse—Wishart (NIW) priors for covariance stabilization, a graph-Laplacian structure over the feature space to capture geometric relations among features, a uniform noise component to absorb outliers, and an adaptive split–merge strategy that refines cluster boundaries. These modules are coupled within a single MAP–EM procedure. The noise component modifies the E-step responsibilities by capturing atypical observations, and these updated responsibilities drive the NIW-regularized covariance and the graph-regularized mean updates in the M-step. The split–merge step is accepted only if it improves the penalized objective. The proposed MAP–EM model was evaluated on five synthetic datasets and three benchmark text corpora, namely Reuters-R8, BBC Sports, and the BBC dataset.

**Results:**

On Reuters-R8, the model achieved an Adjusted Rand Index of 0.347 and an accuracy of 0.569, outperforming the variational Bayesian Gaussian mixture model (GMM) (0.317). On the BBC dataset, it achieved the highest Adjusted Rand Index of 0.326 and a Normalized Mutual Information of 0.425 among the methods compared. Formal statistical testing showed that MAP–EM achieved significant positive differences in 45 out of 75 method-level comparisons, with one significant negative comparison. At the run level, MAP–EM obtained higher scores in 891 out of 1,115 valid paired comparisons, corresponding to a win rate of 79.9%. Theoretical analysis further supports the proposed framework by establishing coercivity of the penalized objective, monotone ascent of the MAP–EM iterations, finite termination of the split–merge stage under the stated acceptance criterion, boundedness of the covariance estimates under the normal–inverse–Wishart prior, local R-linear convergence of the MAP–EM iterations after model-order stabilization, eigenvalue bounds for the NIW covariance estimator, a condition-number bound for the penalized mean update, and a perturbation bound for the graph-regularized mean update.

**Discussion:**

The proposed MAP–EM framework provides a stable, structure-aware clustering approach for high-dimensional text data. Experimental results indicate that its advantages are most evident on datasets with noise, overlapping clusters, and well-connected feature graphs, rather than across all clustering scenarios.

## 1 Introduction

Gaussian mixture models (GMMs) estimated by the Expectation–Maximization (EM) algorithm remain among the most widely used methods for clustering and density estimation. Their appeal lies in their probabilistic interpretation, relative ease of implementation, and ability to produce soft rather than hard cluster assignments (Dempster et al., 1977; Fraley and Raftery, 2002; McLachlan, 2000). The alternating E- and M-step structure also provides an efficient mechanism for maximizing the likelihood in latent-variable models, supporting the broad use of EM in areas such as bioinformatics, imaging, remote sensing, and text analytics.

Despite these advantages, standard GMM–EM has important limitations for modern, complex datasets. Its performance depends strongly on initialization and may converge to poor local optima (Redner and Walker, 1984). Covariance degeneracy is another persistent problem in which a component collapses onto a small set of observations, causing the likelihood to diverge and yielding unstable or uninformative clusters (Bishop, 2006). Furthermore, the classical formulation assumes independently sampled observations and does not directly incorporate structural information such as graph, spatial, or manifold relationships among samples or features (Belkin and Niyogi, 2001; Jia et al., 2014; He et al., 2010).

Outliers pose an additional challenge because a small number of atypical observations can distort Gaussian components or create spurious clusters. Although robust mixture formulations based on heavy-tailed distributions or explicit noise components have been studied (Peel and McLachlan, 2000; Coretto and Hennig, 2016), these methods are typically developed separately from structural regularization and prior-based stabilization. Determining the number of clusters remains difficult. Information criteria such as the Bayesian information criterion (BIC) and the minimum description length (MDL) are typically applied after EM has converged, rather than being incorporated into the estimation procedure itself (Schwarz, 1978; Figueiredo and Jain, 2002). Likewise, split–merge strategies can improve flexibility (Ueda et al., 2000; Ueda and Nakano, 1998), but usually operate as external heuristics rather than within a principled MAP framework.

These limitations motivate the development of an EM-based clustering framework that improves stability, incorporates structural information, explicitly handles noise, and adapts the number of clusters during estimation. To address this issue, this study develops a unified penalized MAP–EM framework for document clustering. The framework integrates normal–inverse–Wishart (NIW) prior regularization, graph-based smoothing, a bounded-support noise component, and split–merge adaptation within a single iterative algorithm. The contribution is to unify these elements within a single penalized objective and to develop an algorithm supported by theoretical analysis.

This study develops a unified MAP–EM model for document clustering that integrates NIW prior regularization, structural smoothing, explicit outlier handling, and adaptive model refinement into a single algorithm. The main contributions are as follows.

- A penalized MAP–EM framework is formulated for document clustering by integrating NIW prior regularization, graph-based smoothing, explicit noise handling, and split–merge adaptation.
- A single iterative algorithm is developed that incorporates these components through noise-aware responsibility updates, regularized parameter estimation, and model-order refinement using split–merge moves.
- The integrated formulation is supported by theoretical results establishing coercivity of the penalized objective, monotone ascent of the MAP–EM iterations, well-posed blockwise M-step updates, finite termination of the split–merge stage, local R-linear convergence after model-order stabilization, and stability properties of the NIW covariance estimator, graph-regularized mean update, and noise responsibility.
- The proposed framework is extensively evaluated on synthetic and benchmark document datasets using ablation studies, parameter sensitivity analysis, statistical validation, scalability analysis, and clustering performance evaluation.

Taken together, these elements extend EM in a regularized form and address covariance degeneracy, structural dependence, noise sensitivity, and cluster-number adaptation.

The remainder of this study is organized as follows. Section 2 reviews the literature on Gaussian mixture models, regularized EM algorithms, graph-based clustering, robust mixture modeling, and model selection techniques. Section 3 introduces the preliminaries and notation used throughout the study. Section 4 presents the proposed MAP–EM framework, including the penalized objective, regularized parameter updates, bounded-support noise component, split–merge refinement strategy, and computational complexity analysis. Section 5 develops the theoretical foundation of the proposed method by establishing coercivity of the penalized objective, monotone ascent, blockwise well-posedness, local R-linear convergence, condition-number analysis, eigenvalue bounds, perturbation analysis, bounded-noise responsibilities, and the resulting practical guarantees. Section 6 reports the experimental evaluation, including ablation studies, parameter sensitivity analysis, scalability, statistical validation, robustness analysis, and benchmark document clustering experiments. Finally, Section 7 concludes the study and discusses future research directions.

## 2 Related work

The expectation–maximization (EM) algorithm is a standard method for parameter estimation in models with unobserved or hidden variables. Its theoretical foundations were established by Dempster et al. (1977), and its convergence properties were later refined by Wu (1983) and Redner and Walker (1984). Model-based clustering was systematized by Fraley and Raftery (2002), and Celeux and Govaert (1995) introduced a family of parsimonious Gaussian covariance parameterizations. Comprehensive expositions by McLachlan (2000) and Bishop (2006) consolidate these developments and present widely adopted formulations of Gaussian mixture models (GMMs).

A well-known drawback of standard EM is its sensitivity to initialization, tendency to converge to local maxima, and risk of likelihood degeneracy. Robust variants, such as mixtures of *t*-distributions (Peel and McLachlan, 2000) and robust improper likelihood approaches (Coretto and Hennig, 2016), have been proposed to mitigate these issues. Regularization for high-dimensional mixtures was explored by Ruan et al. (2011). These approaches typically address robustness or regularization without jointly incorporating structural priors and adaptive model selection.

Recent theoretical studies have deepened the analysis of the EM algorithm using modern analytical tools. In particular, Balakrishnan et al. (2017) established non-asymptotic guarantees, and Yi and Caramanis (2015) developed a unified framework for regularized EM in high-dimensional latent-variable models. Jin et al. (2016) analyzed the geometry of the GMM likelihood surface, and Dwivedi et al. (2020) established sharp convergence rates for weakly identifiable models. The regularized EM algorithm of Houdouin et al. (2023) offers additional insight into stabilization in high-dimensional settings.

Incorporating structural information into mixture models has become increasingly important. Earlier research on manifold learning and spectral methods, such as Laplacian eigenmaps (Belkin and Niyogi, 2001) and spectral clustering surveys (Jia et al., 2014), established the foundation for graph-based learning. Simplified and hierarchical representations of mixtures of exponential families have also been studied (Garcia and Nielsen, 2010). More recently, graph neural network architectures, including spectral CNNs (Defferrard et al., 2016) and graph convolutional networks (Kipf and Welling, 2017), have underscored the practical importance of Laplacian-based operators on graphs. Laplacian-regularized Gaussian mixture models have also been developed for clustering (He et al., 2010), while related graph-regularized clustering methods have used sparse graph constructions such as L1-graphs (Zheng et al., 2014). Manifold regularization has also been incorporated into deep generative models, demonstrating how geometric structure can inform generative modeling for complex scientific data (Li et al., 2021).

Outlier robustness in mixture modeling has been addressed through heavy-tailed component distributions and explicit robust likelihood formulations. For example, Peel and McLachlan (2000) introduced robust mixture modeling using the *t* distribution, while Coretto and Hennig (2016) developed robust improper maximum-likelihood methods for Gaussian clustering. High-dimensional robustness and parsimony have also been studied using sparse covariance mixture formulations (Fop et al., 2019) and variable-selection methods for model-based clustering (Fop and Murphy, 2018).

Selecting the number of mixture components is another long-standing challenge. Classical information criteria such as BIC (Schwarz, 1978) remain widely used, whereas penalized-likelihood approaches such as that of Figueiredo and Jain (2002) offer a parameter-shrinkage perspective. Beyond information criteria and penalized likelihood, Fang and Wang (2012) have also explored bootstrap-based selection of the number of clusters. Deterministic annealing (Ueda and Nakano, 1998) and split–merge EM methods (Ueda et al., 2000) provide local structural refinements that improve exploration of the likelihood surface. Bayesian sparse finite mixtures as defined by Malsiner-Walli et al. (2016) and consistency results for order estimation by Gassiat and Van Handel (2012) provide additional foundations for model-order selection. Early applications of GMMs to low-level vision include skin-color modeling for image and video databases (Yang and Ahuja, 1998).

Recent studies have underscored the importance of graph construction and structure learning in clustering. Berahmand et al. provided a comprehensive survey of spectral clustering with graph structure learning, classifying existing approaches into pairwise graphs, anchor graphs, and hypergraphs, as well as fixed and adaptively learned graph structures. Their review highlights that clustering performance is strongly influenced by the quality of the underlying similarity graph, particularly in high-dimensional and large-scale settings (Berahmand et al., 2025a). Berahmand et al. also proposed an attributed spectral clustering method based on a parameter-free affinity matrix that jointly incorporates network topology and node attribute information via biased random walks. This approach avoids manual tuning of the affinity parameter and improves the representation of structural and attribute similarities in attributed networks (Berahmand et al., 2022). More recently, the OA2H-SP method introduced a one-step anchor-adaptive hypergraph spectral clustering framework in which anchor relationships, hypergraph structure, and cluster assignments are learned jointly. The method is designed to capture higher-order relationships while reducing the computational burden associated with conventional spectral clustering on large datasets (Berahmand et al., 2025b). These developments demonstrate the growing role of adaptive graph and hypergraph learning in clustering. Conversely, the present study neither performs spectral partitioning nor learns a sample-level affinity graph. Instead, it incorporates a graph Laplacian over the feature space as a regularization term within a probabilistic MAP–EM mixture model. The feature graph therefore serves as prior structural information that encourages related features to have smoother cluster-mean representations, while the final cluster assignments are obtained through posterior mixture responsibilities.

### 2.1 Research gap

Although the literature offers many extensions to GMM–EM, these developments remain largely independent of each other. Bayesian formulations using normal–inverse–Wishart (NIW) priors provide stable covariance estimates and prevent degeneracy, but they do not incorporate structural relationships among features or samples (McLachlan, 2000; Bishop, 2006). Graph-regularized mixture models, on the other hand, encode geometric or network structures (Belkin and Niyogi, 2001), yet they remain sensitive to noise and do not address the problem of covariance collapse, which is common in high-dimensional mixtures. Robust mixture modeling introduces noise components or heavy-tailed distributions (Peel and McLachlan, 2000; Coretto and Hennig, 2016). However, these approaches are typically developed without any structural regularization or prior-based information.

A complementary line of study focuses on selecting the number of clusters using penalized-likelihood and split-merge strategies (Schwarz, 1978; Figueiredo and Jain, 2002; Ueda et al., 2000). However, these methods generally operate as external mechanisms and do not integrate naturally with regularized EM formulations or graph-based constraints.

Thus, most existing methods treat stability, structural information, robustness, and model selection separately. But these challenges often appear together in modern datasets. This motivates an EM-based iterative and unified algorithm for modern datasets. This iterative algorithm preserves the advantages of classical mixture models while incorporating prior regularization, structural smoothing, explicit noise handling, and adaptive model refinement in a single formulation.

## 3 Preliminaries and notation

Let the dataset


$$X={\left\{x_{i}\right\}}_{i=1}^{N}\subset {\mathbb{R}}^{d},$$

be a collection of *N* samples in *d*-dimensional space.

A *K*-component Gaussian mixture is parameterized as


$$\Theta ={\left\{\left(\pi_{k},\mu_{k},\Sigma_{k}\right)\right\}}_{k=1}^{K},$$

where π_*k*_≥0 and $\overset{K}{\underset{k=1}{\sum }}\pi_{k}=1$. Each component corresponds to a Gaussian density


$$N\left(x|\mu_{k},\Sigma_{k}\right).$$

For each sample *x*_*i*_, the responsibility of component *k* is denoted as *r*_*ik*_, with weights *w*_*i*_>0 to account for the varying level of importance.

The effective membership count for cluster *k* is


$$N_{k}=\overset{N}{\underset{i=1}{\sum }}w_{i}r_{ik}.$$

The empirical mean of cluster *k* is


$${\bar{x}}_{k}=\frac{1}{N_{k}}\overset{N}{\underset{i=1}{\sum }}w_{i}r_{ik}x_{i},$$

and the empirical scatter matrix is


$$S_{k}=\overset{N}{\underset{i=1}{\sum }}w_{i}r_{ik}\left(x_{i}-{\bar{x}}_{k}\right){\left(x_{i}-{\bar{x}}_{k}\right)}^{⊤}.$$

### 3.1 Graph Laplacian

When the relational structure is known (e.g., feature similarity graphs), it can be encoded as a symmetric positive semidefinite feature-graph Laplacian matrix *L*. The cluster means are penalized by


$$\frac{\gamma }{2}\overset{K}{\underset{k=1}{\sum }}\mu_{k}^{⊤}L\mu_{k},$$

where γ>0 controls the strength of the regularization.

### 3.2 Normal–inverse–Wishart prior

To stabilize the estimation and prevent degeneracy, a conjugate normal–inverse–Wishart (NIW) prior was adopted. For each component *k*,


$$\mu_{k}|\Sigma_{k}\sim N\left(m_{0},\frac{1}{\kappa_{0}}\Sigma_{k}\right),\;\Sigma_{k}\sim \mathrm{IW}\left(S_{0},\nu_{0}\right).$$

This prior function acts as a Bayesian regularizer. This shrinks the means toward *m*_0_ and prevents covariances Σ_*k*_ from collapsing.

### 3.3 Notation

The notations used in the proposed MAP–EM model are tabulated in Table 1.

**Table 1:** Notation and symbols used in the proposed MAP–EM model.

Symbol	Meaning
$X={\left\{x_{i}\right\}}_{i=1}^{N}$	Dataset of *N* samples in ℝ^*d*^
*K*	Number of mixture components
π_*k*_	Mixing proportion of component *k*
μ_*k*_	Mean vector of component *k*
Σ_*k*_	Covariance estimate of component *k*
$\Theta ={\left\{\pi_{k},\mu_{k},\Sigma_{k}\right\}}_{k=1}^{K}$	Model parameters (weights, means, covariances)
*r*_*ik*_	Responsibility of component *k* for sample *i*
*w*_*i*_	Sample weights (default *w*_*i*_ = 1)
*N*_*k*_	Effective membership count of cluster *k*
${\bar{x}}_{k}$	Empirical mean of cluster *k*
*S*_*k*_	Empirical scatter matrix of cluster *k*
*L*	Graph Laplacian matrix over feature space
γ	Graph regularization strength
*m*_0_	Prior mean (NIW)
κ_0_	Scaling factor for NIW mean prior
*S*_0_	Scale matrix for NIW covariance prior
ν_0_	Degrees of freedom for NIW covariance prior
κ_*k*_, ν_*k*_	Posterior NIW parameters
*S*_*k*_	Empirical scatter matrix
$S_{k}^{\prime }$	Posterior scale matrix for component *k*
ℓ(Θ)	Weighted log-likelihood of observed data
*F*(Θ)	MAP–EM objective
J(Θ,K)	Penalized split–merge score
$\tilde{Q}\left(\Theta |\Theta^{\left(t\right)}\right)$	Penalized surrogate
*D*(Θ′||Θ)	Non-negative surrogate discrepancy term
*A*	Adjacency matrix of the feature graph
*D*	Degree matrix of the feature graph
γ	Laplacian regularization parameter
β_noise_	Noise-component scaling parameter
β	Model-complexity penalty parameter
*k*_*NN*_	Neighborhood size in the feature graph
max_moves	Maximum number of split–merge refinements
*m*_*k*_	Posterior mean of component *k* under the NIW prior update
Ω	Bounded support of the uniform noise component
*a*_*j*_, *b*_*j*_	Lower and upper bounds of Ω in the *j*th feature dimension
δ_*j*_	Margin used to expand the support interval in the *j*th dimension
|Ω|	Volume of the support region Ω
*p*_0_(*x*)	Uniform density on Ω
${\tilde{s}}_{0}\left(x\right)$	Scaled uniform noise density
π_0_	Mixing proportion of the noise component
*r*_*i*0_	Noise-component responsibility for sample *i*
λ_min_(Σ_*k*_), λ_max_(Σ_*k*_)	Minimum and maximum eigenvalues of Σ_*k*_
D	Feasible parameter space
M(Θ)	Continuous MAP–EM update mapping
$L_{c}$	Upper-level set {Θ∈D:F(Θ)≥c}
*I*_*c*_	Complete-information Hessian matrix
*I*_*o*_	Observed-information Hessian matrix
$J_{M}\left(\Theta^{*}\right)$	Jacobian of the MAP–EM update mapping evaluated at the stationary point Θ^*^
ρ(·)	Spectral radius of a matrix

## 4 Proposed MAP–EM model

Gaussian mixture clustering is attractive for its probabilistic formulation and soft assignment mechanism. However, these strengths can become less effective in more challenging data. In high-dimensional data, covariance estimates may become ill-conditioned. Covariance estimation also becomes unreliable when some mixture components have a small effective sample size. This can lead to unstable updates and, in extreme cases, degenerate solutions. When clusters overlap, or the data exhibit structural heterogeneity, the classical EM framework does not account for known relationships among features. Thus, it may recover clusters that do not align well with the underlying data structure. Even a small number of atypical observations can distort the Gaussian components or give rise to spurious clusters. Furthermore, an incorrect choice of the number of clusters may lead to over-segmentation or inappropriate merging of distinct groups. These problems become more pronounced when high dimensionality, overlap, noise, and structural dependence occur together, motivating the proposed MAP–EM model.

The proposal is to keep the core idea alternating between soft assignments (E-step) and parameter updates (M-step)—but embed several safeguards and extensions to keep the algorithm well-behaved in practice. To address these limitations, the proposed framework integrates a Bayesian prior for covariance regularization, a structural penalty that preserves known feature relationships, an explicit noise component for robust outlier handling, and a split–merge mechanism that automatically adapts the number of clusters. Together, these components improve the robustness and flexibility of the clustering process.

Instead of maximizing only the likelihood, the objective function maximizes the penalized posterior objective given by


$$\begin{array}{l} F\left(\Theta \right)=\ell \left(\Theta \right)+\log p_{\text{NIW}}\left(\left\{\mu_{k},\Sigma_{k}\right\}\right)-\frac{\gamma }{2}\overset{K}{\underset{k=1}{\sum }}\mu_{k}^{⊤}L\mu_{k}, \end{array}$$ (1)

where


$$\begin{array}{l} \ell \left(\Theta \right)=\overset{N}{\underset{i=1}{\sum }}w_{i}\log\overset{K}{\underset{k=1}{\sum }}\pi_{k}N\left(x_{i}|\mu_{k},\Sigma_{k}\right). \end{array}$$ (2)

The objective in Equation 1, together with the weighted log-likelihood defined in Equation 2, balances three forces: (i) the log-likelihood of the data under the mixture, (ii) graph smoothness via the Laplacian penalty, and (iii) stability through the NIW prior.

Because the priors and regularization terms act directly on the parameters rather than the latent assignments, the E-step remains unchanged from the classical EM and computes the responsibilities *r*_*ik*_ using Equation 3. The M-step, no longer maximizes the standard *Q*-function but instead maximizes the penalized surrogate $\tilde{Q}\left(\Theta |\Theta^{\left(t\right)}\right)=Q\left(\Theta |\Theta^{\left(t\right)}\right)+C\left(\Theta \right)$, where *C*(Θ) encapsulates the NIW prior and the Laplacian penalty. This is equivalent to determining the MAP estimate of the parameters given the current responsibilities. The updates for the mixing proportions π_*k*_, means μ_*k*_, and covariances Σ_*k*_ derived are closed-form or iterative solutions to this penalized maximization problem.

The responsibilities are given by,


$$\begin{array}{l} r_{ik}=\frac{\pi_{k}N\left(x_{i}|\mu_{k},\Sigma_{k}\right)}{\overset{K}{\underset{j=1}{\sum }}\pi_{j}N\left(x_{i}|\mu_{j},\Sigma_{j}\right)}. \end{array}$$ (3)

These responsibilities yield effective counts *N*_*k*_, empirical means ${\bar{x}}_{k}$, and scatters *S*_*k*_.

Mixing proportions are updated in the standard way given by,


$$\begin{array}{l} \pi_{k}=\frac{N_{k}}{\underset{j}{\sum }N_{j}}. \end{array}$$ (4)

The mixing-proportion update in Equation 4 normalizes the effective membership count of each component by the total effective membership. In classical EM, $\mu_{k}={\bar{x}}_{k}$. With an NIW prior, μ_*k*_ is the weighted average of the prior mean *m*_0_ and the empirical mean. The graph Laplacian adds a third pull, thereby enforcing structural smoothness. Collecting all terms that depend on the cluster mean μ, the objective function is given by


$$\begin{array}{l} \begin{array}{cc} Q\left(\mu \right) & =\frac{N_{k}}{2}{\left(\mu -{\bar{x}}_{k}\right)}^{⊤}\Sigma_{k}^{-1}\left(\mu -{\bar{x}}_{k}\right)\\ +\frac{\kappa_{0}}{2}{\left(\mu -m_{0}\right)}^{⊤}\Sigma_{k}^{-1}\left(\mu -m_{0}\right)+\frac{\gamma }{2}\mu^{⊤}L\mu . \end{array} \end{array}$$ (5)

Setting ∇_μ_*Q* = 0 yields the following first-order optimality condition,


$$\begin{array}{l} \left(N_{k}+\kappa_{0}\right)\Sigma_{k}^{-1}+\gamma L\mu_{k}=\Sigma_{k}^{-1}\left(N_{k}{\bar{x}}_{k}+\kappa_{0}m_{0}\right), \end{array}$$ (6)

The resulting linear system balances three sources of information, namely the data likelihood, the NIW prior, and the graph smoothness penalty. The coefficient matrix on the left combines the effective sample count *N*_*k*_, prior strength κ_0_, and graph regularization γ*L*. The right-hand side blends the empirical mean ${\bar{x}}_{k}$ with the prior mean *m*_0_. The update interpolates between data-driven estimates and prior knowledge while respecting the feature-space structure encoded in *L*.

Equation 5 requires solving a symmetric positive-definite linear system. The Laplacian-regularized mean update is solved using the preconditioned conjugate gradient (PCG) method, which exploits the sparsity of *L* and the improved conditioning of Σ_*k*_ under NIW priors. This ensures stable and efficient computation even in moderately high-dimensional settings.

The graph regularization in Equations 5 and 6 is defined on a feature graph. In the implementation, each node corresponds to a feature term from the TF–IDF representation, and pairwise affinities are computed from feature-occurrence patterns across documents. A weighted *k*-nearest-neighbor feature graph is constructed, symmetrized, and converted to the Laplacian matrix *L* = *D*−*A*, where *A* is the adjacency matrix and *D* is the corresponding degree matrix. The graph is built once before the MAP–EM iterations and kept fixed throughout the optimization of the penalized objective. The graph regularization strength is controlled by the parameter γ appearing in Equations 5 and 6.

Without regularization, the EM covariances can collapse, which tends the likelihood to infinity. The NIW prior mitigates this issue by augmenting the empirical scatter matrix with prior evidence and effectively adding pseudo–observations that stabilize the covariance estimation.

After observing *N*_*k*_ points,


$$\begin{array}{ll} \kappa_{k} & =\kappa_{0}+N_{k}, \end{array}$$ (7)

$$\begin{array}{ll} \nu_{k} & =\nu_{0}+N_{k}, \end{array}$$ (8)

$$\begin{array}{ll} m_{k} & =\frac{\kappa_{0}m_{0}+N_{k}{\bar{x}}_{k}}{\kappa_{0}+N_{k}}. \end{array}$$ (9)

Equations 7–9 define the posterior NIW parameters after incorporating the effective membership *N*_*k*_. The posterior scale matrix $S_{k}^{\prime }$ merges three distinct sources of second-moment information,


$$\begin{array}{l} S_{k}^{\prime }=S_{0}+S_{k}+\frac{\kappa_{0}N_{k}}{\kappa_{0}+N_{k}}\left({\bar{x}}_{k}-m_{0}\right){\left({\bar{x}}_{k}-m_{0}\right)}^{⊤}. \end{array}$$ (10)

In Equation 10, *S*_0_ is the prior scale matrix, *S*_*k*_ is the empirical scatter matrix, and the third term accounts for the discrepancy between the empirical mean ${\bar{x}}_{k}$ and the prior mean *m*_0_, weighted by the relative influence of the prior κ_0_/(κ_0_+*N*_*k*_).

This composite update ensures that the covariance estimate remains positive definite and well-conditioned even when the effective sample size *N*_*k*_ is small.

The resulting MAP estimate for the covariance is


$$\begin{array}{l} \Sigma_{k}=\frac{S_{k}^{\prime }}{\nu_{k}+d+1}. \end{array}$$ (11)

The covariance estimate in Equation 11 remains positive definite even when *N*_*k*_ is small.

To account for atypical observations, an additional uniform noise component is included in the mixture model. This component absorbs outliers and isolated points that are poorly explained by the Gaussian clusters, thereby reducing their influence on the Gaussian parameter estimates and limiting the formation of spurious small clusters. To ensure that the contribution of this component is well-defined, its support Ω is restricted to a bounded region of the data space. In practice, instead of computing an exact convex hull, we use a hyper-rectangular approximation and define it by


$$p_{0}(x)=\{\begin{array}{ll} \frac{1}{\left|\Omega \right|}, & x\in \Omega ,\\ 0, & x\notin \Omega , \end{array}$$ (12)

where Ω is a bounded region covering the observed data and is given by


$$\begin{array}{l} \Omega =\overset{d}{\underset{j=1}{\prod }}\left[a_{j}-\delta_{j},b_{j}+\delta_{j}\right], \end{array}$$ (13)

with $a_{j}=\underset{i}{\min}x_{ij}$, $b_{j}=\underset{i}{\max}x_{ij}$, and δ_*j*_>0 a small margin in the *j*th feature dimension. This construction avoids explicit convex-hull computation, ensures that |Ω| < ∞, and keeps the uniform noise component properly defined within the MAP–EM procedure. The corresponding support volume is


$$\begin{array}{l} |\Omega |=\overset{d}{\underset{j=1}{\prod }}\left(\left(b_{j}-a_{j}\right)+2\delta_{j}\right). \end{array}$$ (14)

Equations 13 and 14 define the bounded hyper-rectangular support and its corresponding volume, respectively. To regulate the contribution of the uniform noise component, we introduce a hyperparameter β_noise_>0 and define the scaled noise term


$$\begin{array}{l} {\tilde{s}}_{0}\left(x\right)=\beta_{\text{noise}}p_{0}\left(x\right), \end{array}$$ (15)

where *p*_0_(*x*) is the bounded-support uniform density in Equation 12. The scaled noise density defined in Equation 15 controls the contribution of the uniform noise component through β_noise_. Smaller values of β_noise_ make the noise component more selective, so that only points with very low Gaussian support are assigned to it, whereas larger values increase the tendency to absorb borderline observations as noise. Here, π_0_ denotes the mixing weight of the noise component, while β_noise_ is a fixed tuning parameter that controls its competitiveness in the responsibility update.

Accordingly, for an observation *x*_*i*_, the posterior responsibility of the noise component is given by


$$\begin{array}{l} r_{i0}=\frac{\pi_{0}{\tilde{s}}_{0}\left(x_{i}\right)}{\pi_{0}{\tilde{s}}_{0}\left(x_{i}\right)+\overset{K}{\underset{k=1}{\sum }}\pi_{k}N\left(x_{i}|\mu_{k},\Sigma_{k}\right)}. \end{array}$$ (16)

As shown in Equation 16, for points located near a cluster center, the Gaussian terms dominate and *r*_*i*0_ remains close to zero. Conversely, for observations far from all cluster centers, the Gaussian densities become small, and the noise responsibility increases.

To illustrate the role of the noise component, consider a mixture with two Gaussian components and one uniform noise component. Suppose the mixing weight of the noise component is π_0_ = 0.10 and the scaled uniform density is ${\tilde{s}}_{0}\left(x\right)=5\times 10^{-4}$. For an observation near a cluster center, the weighted Gaussian likelihood is much larger than the weighted noise contribution, yielding a negligible noise responsibility (e.g., $r_{i0}\approx 3\times 10^{-4}$). Conversely, for an observation located far from all cluster centers, the Gaussian likelihoods become comparable to the weighted uniform density, producing a substantially larger noise responsibility (e.g., *r*_*i*0_≈0.27). Thus, observations that are well-explained by the Gaussian components are assigned almost entirely to those components, whereas isolated or atypical observations receive greater responsibility from the uniform noise component. This mechanism reduces the influence of outliers on the Gaussian parameter estimates without altering the probabilistic EM framework.

Model selection was incorporated using a split–merge scheme. The penalized score derived from the MAP objective is defined as,


$$\begin{array}{l} J\left(\Theta ,K\right)=F\left(\Theta \right)-\text{pen}\left(K\right), \end{array}$$ (17)

where


$$\begin{array}{l} pen\left(K\right)=\frac{\beta }{2}\left(\#\text{free parameters}\right)\log N. \end{array}$$

The penalized score in Equation 17 is used to evaluate the split-merge proposals. A component is split if it is overly anisotropic, merged if it is highly overlapping, and accepted only if ΔJ>0. Each accepted move is followed by rerunning the MAP–EM to ensure convergence. Because J strictly increases and only finitely many partitions exist, termination is guaranteed. In the implementation, the number of refinement moves is capped by max_moves, so the split–merge wrapper terminates in finitely many steps.

Here, β>0 controls the strength of the complexity penalty. Setting β = 1 yields the standard BIC penalty $\frac{1}{2}p\log N$, where *p* is the number of free parameters. For comparison, an AIC-type penalty would use *p* without the log*N* factor. In the experiments, this default choice was used. In general, β can be tuned empirically to balance the fit quality and model simplicity in the split–merge process.

**Figure 1:**
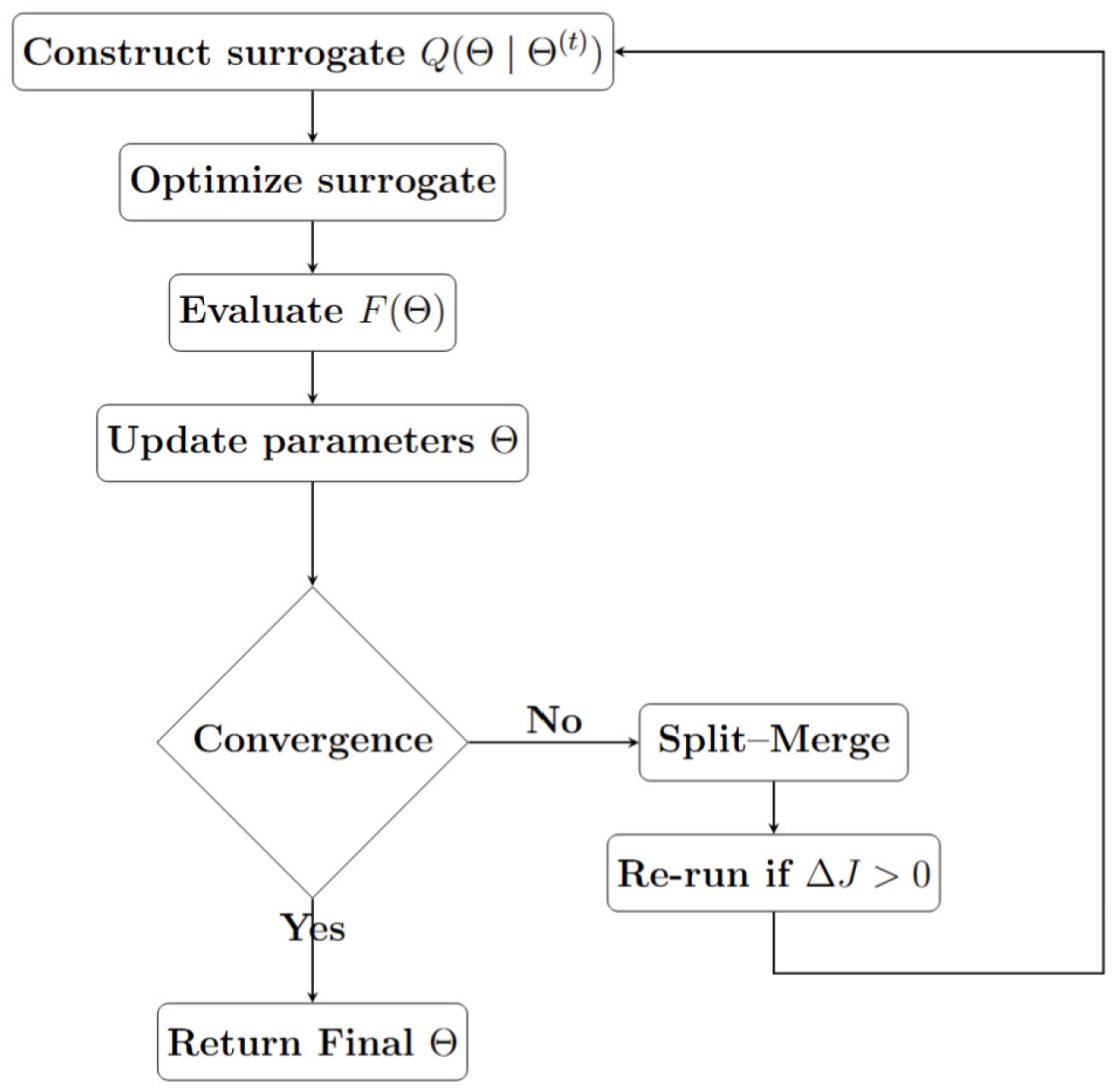
Work flow of the proposed MAP–EM model.

Figure 1 depicts the workflow of the maximum a posteriori expectation–maximization (MAP–EM) algorithm with the integrated split–merge refinement process.

### 4.1 Computational complexity

Let *N* be the number of data points, *d* the feature dimension, *K* the number of components, and nnz(*L*) the number of non-zeros in the feature graph Laplacian. A single MAP–EM iteration has the following cost:

E-step: Evaluating Gaussian responsibilities costs *O*(*NKd*^2^) for full covariances (using precomputed Cholesky factors of Σ_*k*_), reducible to *O*(*NKd*) for diagonal covariances.

Mean updates: For each component *K*, the NIW+laplacian M-step for μ_*k*_ reduces to solving a sparse linear system in the *d*-dimensional feature space, with a coefficient matrix that combines a diagonal term and the feature-graph Laplacian *L*. Solving this system by conjugate gradients costs *O*(nnz(*L*)*T*_CG_) per component, or *O*(*K*nnz(*L*)*T*_CG_) overall, where *T*_CG_ is the number of iterations in the algorithm.

Covariance updates: NIW posteriors admit closed-form updates based on sufficient statistics; computing and factorizing each Σ_*k*_ is *O*(*d*^3^), so the total cost is *O*(*Kd*^3^).

Split–merge: Occasional split–merge proposals require *O*(*K*^2^) scoring of candidate pairs, followed by warm-started re-optimization of the affected components.

## 5 Theoretical foundation

In this section, the theoretical foundations of the proposed MAP–EM algorithm are presented. The method is interpreted within an expectation–maximization majorization–minimization (EM/MM) framework. Then, convergence and stability guarantees are established, highlighting the roles of the normal–inverse–Wishart (NIW) priors and the Laplacian regularization. Let


$$F\left(\Theta \right)=\log p\left(X|\Theta \right)+\log p_{\text{NIW}}\left(\left\{\mu_{k},\Sigma_{k}\right\}\right)-\frac{\gamma }{2}\overset{K}{\underset{k=1}{\sum }}\mu_{k}^{⊤}L\mu_{k}.$$

denote the penalized log-posterior, where $\Theta ={\left\{\pi_{k},\mu_{k},\Sigma_{k}\right\}}_{k=1}^{K}$. As in standard EM, the complete-data construction yields a surrogate $\tilde{Q}\left(\Theta^{\prime }|\Theta \right)$ satisfying


$$\begin{array}{l} F\left(\Theta^{\prime }\right)=\tilde{Q}\left(\Theta^{\prime }|\Theta \right)+D\left(\Theta^{\prime }||\Theta \right), \end{array}$$

where *D*(Θ′||Θ)≥0 denotes the non-negative discrepancy between the objective *F*(Θ′) and the surrogate $\tilde{Q}\left(\Theta^{\prime }|\Theta \right)$, and *D*(Θ||Θ) = 0. Hence $\tilde{Q}\left(\Theta^{\prime }|\Theta \right)\leq F\left(\Theta^{\prime }\right)$ for all Θ′, with equality at Θ′ = Θ.

### 5.1 Assumptions

The following assumptions are used in the convergence analysis.

(A1) ν_0_>*d*−1, so that the inverse-Wishart prior is proper.

(A2) *S*_0_≻0, so that the NIW scale matrix is strictly positive definite.

(A3) *L*≽0 and γ≥0.

(A4) The feasible parameter space satisfies Σ_*k*_≽ϵ*I* for some ϵ>0.

(A5) Each M-step is solved exactly, or the inexact update gives a non-decreasing surrogate value.

**Theorem 1** (Coercivity of the penalized objective). *Assume (A1)–(A4). Let $\left\{\Theta^{\left(n\right)}\right\}\subset D$ be any sequence that leaves every compact subset of the feasible parameter space D. Then *F*(Θ^(*n*)^) → −∞. Consequently, every upper-level set $L_{c}=\left\{\Theta \in D:\;F\left(\Theta \right)\geq c\right\}$ is compact.*

*Proof:* Let $\Theta^{\left(n\right)}={\left\{\left(\pi_{k}^{\left(n\right)},\mu_{k}^{\left(n\right)},\Sigma_{k}^{\left(n\right)}\right)\right\}}_{k=1}^{K}.$ Since the mixing weights lie in the probability simplex, the sequence can leave every compact subset of D only if, for some component *k*, at least one of the following occurs along a subsequence:


$$||\mu_{k}^{\left(n\right)}||\to \infty ,\;\lambda_{\max}\left(\Sigma_{k}^{\left(n\right)}\right)\to \infty ,\;\lambda_{\min}\left(\Sigma_{k}^{\left(n\right)}\right)\to 0.$$

These cases were considered separately.

*Case 1:* $\lambda_{\max}\left(\Sigma_{k}^{\left(n\right)}\right)\to \infty$.

The inverse–Wishart part of the NIW prior contributes, up to an additive constant independent of Σ_*k*_,


$$-\frac{\nu_{0}+d+1}{2}\log\det\left(\Sigma_{k}\right)-\frac{1}{2}\mathrm{tr}\left(S_{0}\Sigma_{k}^{-1}\right)$$

to the objective *F*(Θ). If $\lambda_{\max}\left(\Sigma_{k}^{\left(n\right)}\right)\to \infty$, then $\log\det\left(\overset{\left(n\right)}{\underset{k}{\Sigma }}\right)\to \infty$, so the first term tends to −∞. Hence *F*(Θ^(*n*)^) → −∞.

*Case 2:* $\lambda_{\min}\left(\Sigma_{k}^{\left(n\right)}\right)\to 0$.

Because *S*_0_≻0 by assumption (A2), there exists a constant *m*_0_>0 such that *S*_0_≽*m*_0_*I*. Therefore, $\mathrm{tr}\left(S_{0}\Sigma_{k}^{-1}\right)\geq m_{0}\mathrm{tr}\left(\Sigma_{k}^{-1}\right).$ If $\lambda_{\min}\left(\Sigma_{k}^{\left(n\right)}\right)\to 0$, then $\mathrm{tr}\left({\left(\Sigma_{k}^{\left(n\right)}\right)}^{-1}\right)\to \infty$, and hence $\mathrm{tr}\left(S_{0}{\left(\Sigma_{k}^{\left(n\right)}\right)}^{-1}\right)\to \infty .$ Thus the barrier term from the inverse–Wishart prior forces *F*(Θ^(*n*)^) → −∞.

*Case 3:* $||\mu_{k}^{\left(n\right)}||\to \infty$, while the eigenvalues of $\Sigma_{k}^{\left(n\right)}$ remain uniformly bounded above and below.

Then there exist constants 0 < *m* ≤ *M* < ∞ such that $mI≼\Sigma_{k}^{\left(n\right)}≼MI$ for all sufficiently large *n*. Hence ${\left(\Sigma_{k}^{\left(n\right)}\right)}^{-1}≽\frac{1}{M}I.$ The Gaussian part of the NIW prior contributes, up to an additive constant,


$$-\frac{\kappa_{0}}{2}{\left(\mu_{k}-m_{0}\right)}^{⊤}\Sigma_{k}^{-1}\left(\mu_{k}-m_{0}\right)$$

to *F*(Θ). Therefore,


$$-\frac{\kappa_{0}}{2}{\left(\mu_{k}^{\left(n\right)}-m_{0}\right)}^{⊤}{\left(\Sigma_{k}^{\left(n\right)}\right)}^{-1}\left(\mu_{k}^{\left(n\right)}-m_{0}\right)\leq -\frac{\kappa_{0}}{2M}||\mu_{k}^{\left(n\right)}-m_{0}|{|}^{2}.$$

Since $||\mu_{k}^{\left(n\right)}||\to \infty$, the right-hand side tends to −∞, and thus *F*(Θ^(*n*)^) → −∞. These three cases exhaust all possible ways in which a sequence can leave every compact subset of D. Hence *F* is coercive on D.

Now let *c*∈ℝ. By coercivity, the upper-level set $L_{c}=\left\{\Theta \in D:\;F\left(\Theta \right)\geq c\right\}$ is bounded. Let $\Theta^{\left(n\right)}\in L_{c}$ and suppose that Θ^(*n*)^ → Θ^*^ in the ambient parameter space. If $\Theta^{*}\notin D$, then some covariance matrix must satisfy $\lambda_{\min}\left(\Sigma_{k}^{*}\right)=0$, which is impossible for a sequence staying inside the upper-level set by the argument of Case 2. Hence $\Theta^{*}\in D$. Since *F* is continuous on D, $F\left(\Theta^{*}\right)=\underset{n\to \infty }{\lim}F\left(\Theta^{\left(n\right)}\right)\geq c,$ so $\Theta^{*}\in L_{c}$. Therefore $L_{c}$ is closed and bounded, hence compact.

**Theorem 2** (Monotone ascent of the penalized objective). *Under assumptions (A1)–(A5), the MAP–EM updates*


$$\Theta^{\left(t+1\right)}=\arg\underset{\Theta }{\max}\tilde{Q}\left(\Theta |\Theta^{\left(t\right)}\right)$$

*satisfy*


$$F\left(\Theta^{\left(t+1\right)}\right)\geq F\left(\Theta^{\left(t\right)}\right)\;\text{for all}t\geq 0.$$

*Moreover, any accumulation point Θ of* ${\left\{\Theta^{\left(t\right)}\right\}}_{t\geq 0}$ is a stationary point of *F*.

*Proof:* The usual EM identity gives, up to a constant independent of Θ,


$$\begin{array}{lll} \log p\left(X|\Theta \right) & = & Q\left(\Theta |\Theta^{\left(t\right)}\right)-\text{KL}p\left(Z|X,\Theta^{\left(t\right)}\right)||p\left(Z|X,\Theta \right)\\ +\text{const}\left(\Theta^{\left(t\right)}\right), \end{array}$$

therefore, log*p*(*X*∣Θ)≥*Q*(Θ∣Θ^(*t*)^) with equality at Θ = Θ^(*t*)^. Adding the penalty


$$C\left(\Theta \right)=\log p_{\text{NIW}}\left(\left\{\mu_{k},\Sigma_{k}\right\}\right)-\frac{\gamma }{2}\overset{K}{\underset{k=1}{\sum }}\mu_{k}^{⊤}L\mu_{k}$$

and defining $\tilde{Q}\left(\Theta |\Theta^{\left(t\right)}\right)=Q\left(\Theta |\Theta^{\left(t\right)}\right)+C\left(\Theta \right)$, we obtain


$$F\left(\Theta \right)=\log p\left(X|\Theta \right)+C\left(\Theta \right)\geq \tilde{Q}\left(\Theta |\Theta^{\left(t\right)}\right),$$

with equality at Θ = Θ^(*t*)^.

By assumption (A5), each M-step produces $\tilde{Q}\left(\Theta^{\left(t+1\right)}|\Theta^{\left(t\right)}\right)\geq \tilde{Q}\left(\Theta^{\left(t\right)}|\Theta^{\left(t\right)}\right)$, hence


$$F\left(\Theta^{\left(t+1\right)}\right)\geq \tilde{Q}\left(\Theta^{\left(t+1\right)}|\Theta^{\left(t\right)}\right)\geq \tilde{Q}\left(\Theta^{\left(t\right)}|\Theta^{\left(t\right)}\right)=F\left(\Theta^{\left(t\right)}\right).$$

This proves a monotone ascent.

** Remark 5.1**. Under (A1)–(A4), Theorem 1 shows that the upper-level sets of *F* are compact. Therefore, the MAP–EM iterates {Θ^(*t*)^} remain in a compact subset of the feasible domain, so accumulation points exist, and the monotone sequence {*F*(Θ^(*t*)^)} converges. In this setting, standard MM arguments suggest that any accumulation point Θ^*^ is stationary for *F* in the usual constrained sense.

** Theorem 3** (Blockwise concavity and well-posed M–step updates). *Assume (A1)–(A4). Fix the responsibilities {*r*_*ik*_} and suppose that the corresponding effective counts satisfy *N*_*k*_>0 for all *k*. Then, with all remaining blocks held fixed, the penalized surrogate $\tilde{Q}\left(\Theta |\Theta^{\left(t\right)}\right)$ is strictly concave in the mixing proportions π over the simplex and strictly concave in each mean block μ_*k*_. For each covariance block Σ_*k*_, the associated maximization subproblem over its feasible domain is well-posed and admits a unique solution. Consequently, every single-block M–step subproblem has a unique optimizer, and the corresponding blockwise updates are well-defined.*

*Proof:* For fixed responsibilities, the surrogate consists of the expected complete-data log-likelihood together with the NIW prior terms and the Laplacian penalty. We examine one block at a time.

*Mixing proportions*. The π-dependent part of the surrogate is $\overset{K}{\underset{k=1}{\sum }}N_{k}\log\pi_{k},$ subject to the simplex constraints π_*k*_≥0 and $\overset{K}{\underset{k=1}{\sum }}\pi_{k}=1$. Since *N*_*k*_>0 for all *k*, this function is strictly concave on the interior of the simplex. Hence the mixing-weight update has a unique maximizer.

*Mean block*. Now fix *k*, and hold all other parameters fixed. The terms involving μ_*k*_ come from the Gaussian complete-data log-likelihood, the NIW mean prior, and the Laplacian penalty. Collecting these terms gives a quadratic function of μ_*k*_ with Hessian $-\left(\left(N_{k}+\kappa_{0}\right)\Sigma_{k}^{-1}+\gamma L\right).$ By (A3) and (A4), one has $\Sigma_{k}^{-1}≻0$, *L*≽0, *N*_*k*_+κ_0_>0, and γ≥0. Therefore $\left(N_{k}+\kappa_{0}\right)\Sigma_{k}^{-1}+\gamma L≻0$, so the Hessian is negative definite. Hence, the surrogate is strictly concave in μ_*k*_, and the corresponding block update has a unique solution.

*Covariance block*. Finally, fix *k* and hold the remaining parameters fixed. Up to an additive constant, the Σ_*k*_-dependent part of the surrogate has the form


$$-\frac{\alpha_{k}}{2}\log\det\left(\Sigma_{k}\right)-\frac{1}{2}\mathrm{tr}\left(A_{k}\Sigma_{k}^{-1}\right),$$

where α_*k*_>0 and *A*_*k*_≻0 collects the empirical scatter and NIW prior contributions. Under (A1)–(A4), this defines a well-posed maximization problem over the feasible cone Σ_*k*_≽ε*I*. The corresponding first-order condition has a unique solution, so the covariance update is uniquely determined on its feasible domain.

Thus each single-block maximization problem, namely the updates for π, μ_*k*_, and Σ_*k*_ with all other blocks fixed, have a unique optimizer. Therefore, the blockwise M–step updates are well-defined.

** Remark 5.2**. *Theorem 3 establishes blockwise concavity and uniqueness of the single-block M-step updates. However, it does not assert the joint concavity of the full surrogate in (μ_*k*_, Σ_*k*_). This is because the surrogate contains the coupled quadratic term*


$$-\frac{1}{2}\left(N_{k}+\kappa_{0}\right){\left(\mu_{k}-{\tilde{m}}_{k}\right)}^{⊤}\Sigma_{k}^{-1}\left(\mu_{k}-{\tilde{m}}_{k}\right),$$

*which links the mean and covariance blocks through the inverse covariance matrix. Consequently, the surrogate is not separable in (μ_*k*_, Σ_*k*_), and no direct joint concavity claim is made here. For this reason, the theorem is intentionally stated in a blockwise form.*

** Proposition 1** (Finite termination of the split–merge stage). *Let *J*(Θ, *K*) = *F*(Θ)−pen(*K*) denote the penalized objective function. Suppose that every proposed split or merge is accepted only if it strictly increases *J*, and that the number of candidate clusters satisfies *K* ≤ *K*_max_. Then the split–merge stage terminates after finitely many accepted updates.*

*Proof:* By Theorem 1, the iterates remain inside a compact upper-level set of the penalized objective. Since the number of mixture components is bounded by *K*_max_, only finitely many partitions of the data are possible. Moreover, each accepted split or merge produces a strict increase in the objective value *J*. Therefore, no partition can be accepted more than once, and an infinite sequence of accepted updates is impossible. Hence, the split–merge stage terminates after finitely many accepted updates.

** Remark 5.3**. *Proposition 1 establishes only finite termination of the heuristic split–merge stage. It does not imply global optimality, nor does it provide approximation or regret guarantees, which are generally unavailable for split–merge EM algorithms.*

Theoretical results establish coercivity, monotone ascent, bounded covariance behavior, well-posed blockwise updates, and a local linear convergence rate after the split–merge configuration has stabilized.

** Theorem 4**. [Local R-linear convergence of the MAP–EM iterations] Assume (A1)–(A5). After the split–merge stage has terminated, assume that the number of mixture components and the graph structure remain fixed, and let $\Theta^{\left(t+1\right)}=M\left(\Theta^{\left(t\right)}\right)=\arg\underset{\Theta }{\max}\tilde{Q}\left(\Theta |\Theta^{\left(t\right)}\right),$ where $\tilde{Q}$ denotes the penalized MAP–EM surrogate. Let Θ^*^ be an interior strict local maximizer of the penalized objective *F*(Θ) satisfying ∇*F*(Θ^*^) = 0, and define $I_{c}=-\nabla_{11}^{2}\tilde{Q}\left(\Theta^{*}|\Theta^{*}\right),\;I_{o}=-\nabla^{2}F\left(\Theta^{*}\right).$ If 0≺*I*_*o*_≼*I*_*c*_, then the local MAP–EM update mapping satisfies $\rho \left(J_{M}\left(\Theta^{*}\right)\right)<1,$ where ρ(·) denotes the spectral radius and $J_{M}\left(\Theta^{*}\right)=\frac{\partial M\left(\Theta \right)}{\partial \Theta }{|}_{\Theta =\Theta^{*}}$ denote the Jacobian of the local MAP–EM update mapping at the stationary point. Consequently, there exist constants *C*>0, 0 < *r* < 1, and a neighborhood U of Θ^*^ such that, for every initialization $\Theta^{\left(0\right)}\in U$,


$$||\Theta^{\left(t\right)}-\Theta^{*}||\leq Cr^{t}||\Theta^{\left(0\right)}-\Theta^{*}||,\;t=0,1,\ldots .$$

Hence, the MAP–EM iterates converge locally R-linearly to Θ^*^.

*Proof:* After the split–merge stage terminates, the model dimension remains fixed and the MAP–EM iteration defines a local fixed-point mapping Θ^(*t*+1)^ = *M*(Θ^(*t*)^). Using the EM tangency identity $\nabla F\left(\Theta \right)=\nabla_{1}\tilde{Q}\left(\Theta |\Theta \right)$, which extends naturally to the penalized surrogate, together with the first-order optimality condition of the M-step, the Implicit Function Theorem implies that *M* is continuously differentiable in a neighborhood of Θ^*^ (Ortega and Rheinboldt, 1970; Dempster et al., 1977; Wu, 1983). Differentiating the M-step optimality condition at Θ^*^ gives the Jacobian $J_{M}\left(\Theta^{*}\right)=I-I_{c}^{-1}I_{o}$, which is the classical local EM convergence matrix adapted to the present penalized MAP–EM formulation (Wu, 1983; Meng and Rubin, 1994). Since 0≺*I*_*o*_≼*I*_*c*_, all eigenvalues of $I_{c}^{-1}I_{o}$ belong to (0, 1] by the Loewner ordering for symmetric positive-definite matrices (Horn and Johnson, 2012). Therefore, $\rho \left(J_{M}\left(\Theta^{*}\right)\right)=\rho \left(I-I_{c}^{-1}I_{o}\right)<1.$ Hence, *M* is a local contraction. By the Banach Fixed-Point Theorem (Ortega and Rheinboldt, 1970), there exist constants *C*>0 and 0 < *r* < 1 such that


$$||\Theta^{\left(t\right)}-\Theta^{*}||\leq Cr^{t}||\Theta^{\left(0\right)}-\Theta^{*}||,$$

for all sufficiently large *t*. Therefore, the MAP–EM iterates converge locally R-linearly to Θ^*^.

** Remark 5.4** (Scope of the rate result). *Theorem 4 applies only after the number of Gaussian components and the split–merge configuration are fixed. A split or merge changes the model dimension and therefore cannot be represented by a differentiable self-map on one fixed-dimensional parameter space. The theorem describes the local convergence rate of the continuous MAP–EM re-optimization performed between accepted split–merge moves and after the final model configuration has been selected.*

** Remark 5.5** (Additional local condition). *The condition 0≺*I*_*o*_≼*I*_*c*_ is an additional local regularity assumption and is not implied by Assumptions (A1)–(A5). It corresponds to the classical missing-information principle for EM algorithms and is assumed here for the penalized MAP–EM formulation.*

** Remark 5.6 (Dependence of the convergence rate)**. *The local convergence rate is determined by $\rho \left(I-I_{c}^{-1}I_{o}\right)$. Smaller spectral radii correspond to faster convergence, whereas values close to one indicate slower convergence. In practice, highly overlapping clusters increase the amount of missing information and may therefore slow the local MAP–EM iterations.*

** Lemma 1 (Eigenvalue bounds for the NIW covariance estimator)**. *Let the MAP covariance estimate of the *k*th Gaussian component under the Normal–Inverse–Wishart prior be*


$$\Sigma_{k}=\frac{S_{0}+S_{k}+\frac{\kappa_{0}N_{k}}{\kappa_{0}+N_{k}}\left({\bar{x}}_{k}-m_{0}\right){\left({\bar{x}}_{k}-m_{0}\right)}^{⊤}}{\nu_{0}+N_{k}+d+1},$$

*where* *S*_0_≻0, *S*_*k*_≽0, κ_0_>0, ν_0_>*d*−1.

*Then every eigenvalue of Σ_*k*_ satisfies*


$$\begin{array}{l} \frac{\lambda_{\min}\left(S_{0}\right)+\lambda_{\min}\left(S_{k}\right)}{\nu_{0}+N_{k}+d+1}\leq \lambda_{i}\left(\Sigma_{k}\right)\leq \\ \frac{\lambda_{\max}\left(S_{0}\right)+\lambda_{\max}\left(S_{k}\right)+\frac{\kappa_{0}N_{k}}{\kappa_{0}+N_{k}}||{\bar{x}}_{k}-m_{0}|{|}^{2}}{\nu_{0}+N_{k}+d+1}, \end{array}$$

for *i* = 1, …, *d*.

*Furthermore, for any symmetric positive-definite matrix $\Sigma_{k}^{⋆}$*,


$$|\lambda_{i}\left(\Sigma_{k}\right)-\lambda_{i}\left(\Sigma_{k}^{⋆}\right)|\leq ||\Sigma_{k}-\Sigma_{k}^{⋆}|{|}_{2}.$$

*Consequently, the NIW prior guarantees that the covariance estimate remains positive definite and spectrally stable throughout the MAP–EM iterations.*

*Proof:* The numerator of the NIW covariance estimator is a sum of one positive-definite matrix and two positive-semidefinite matrices. Hence it is positive definite. Applying Weyl's eigenvalue inequalities to the matrix sum (Horn and Johnson, 2012; Theorem 4.3.1) gives the stated lower and upper eigenvalue bounds. Division by the positive scalar ν_0_+*N*_*k*_+*d*+1 preserves these inequalities.

The perturbation bound


$$|\lambda_{i}\left(\Sigma_{k}\right)-\lambda_{i}\left(\Sigma_{k}^{⋆}\right)|\leq ||\Sigma_{k}-\Sigma_{k}^{⋆}|{|}_{2}$$

follows directly from Weyl's eigenvalue perturbation theorem (Horn and Johnson, 2012; Corollary 4.3.3).

Therefore, every covariance estimate remains positive definite, and its eigenvalues remain uniformly bounded under the stated assumptions.

** Remark 5.7**. *Lemma 1 immediately implies*


$$\lambda_{\min}\left(\Sigma_{k}\right)\geq \frac{\lambda_{\min}\left(S_{0}\right)}{\nu_{0}+N_{k}+d+1}>0,$$

*since *S*_*k*_≽0. Consequently, the NIW prior guarantees that every covariance estimate remains strictly positive definite throughout the MAP–EM iterations, thereby preventing the covariance degeneracy commonly encountered in maximum-likelihood estimation of Gaussian mixture models.*

*Furthermore, as the effective cluster size *N*_*k*_ increases, the relative influence of the NIW prior diminishes, and the MAP covariance estimate progressively approaches the empirical covariance estimate. Thus, the NIW prior primarily stabilizes covariance estimation for small or ill-conditioned clusters while having a diminishing effect in the large-sample regime.*

** Proposition 2** (Condition number bound of the penalized mean update). *For the *k*th mixture component, let $H_{\mu_{k}}=\left(N_{k}+\kappa_{0}\right)\Sigma_{k}^{-1}+\gamma L$, where *N*_*k*_≥0, κ_0_>0, Σ_*k*_≻0, γ≥0, and *L*≽0 is the symmetric graph Laplacian. Then *H*_μ_*k*__ is symmetric positive definite, and its spectral condition number satisfies*


$$\kappa_{2}\left(H_{\mu_{k}}\right)\leq \frac{\left(N_{k}+\kappa_{0}\right)\lambda_{\max}\left(\Sigma_{k}^{-1}\right)+\gamma \lambda_{\max}\left(L\right)}{\left(N_{k}+\kappa_{0}\right)\lambda_{\min}\left(\Sigma_{k}^{-1}\right)+\gamma \lambda_{\min}\left(L\right)}$$

*where $\kappa_{2}\left(A\right)=||A|{|}_{2}||A^{-1}|{|}_{2}$ denotes the spectral condition number. Since λ_min_(*L*) = 0 for a graph Laplacian, this reduces to*


$$\kappa_{2}\left(H_{\mu_{k}}\right)\leq \frac{\left(N_{k}+\kappa_{0}\right)\lambda_{\max}\left(\Sigma_{k}^{-1}\right)+\gamma \lambda_{\max}\left(L\right)}{\left(N_{k}+\kappa_{0}\right)\lambda_{\min}\left(\Sigma_{k}^{-1}\right)}.$$

*Moreover, if *mI*≼Σ_*k*_≼*MI*, 0 < *m* ≤ *M* < ∞, and *d*_max_ denotes the maximum weighted degree of the feature graph, then*


$$\kappa_{2}\left(H_{\mu_{k}}\right)\leq \frac{M}{m}+\frac{2\gamma Md_{\max}}{N_{k}+\kappa_{0}}.$$

*Proof:* Since Σ_*k*_≻0, its inverse is symmetric positive definite. Because *N*_*k*_+κ_0_>0, $\left(N_{k}+\kappa_{0}\right)\Sigma_{k}^{-1}≻0.$ Furthermore, *L*≽0 and γ≥0 imply γ*L*≽0. Therefore, $H_{\mu_{k}}=\left(N_{k}+\kappa_{0}\right)\Sigma_{k}^{-1}+\gamma L≻0,$ so the penalized mean-update matrix is symmetric positive definite.

By Weyl's eigenvalue inequalities (Horn and Johnson, 2012),


$$\lambda_{\min}\left(H_{\mu_{k}}\right)\geq \left(N_{k}+\kappa_{0}\right)\lambda_{\min}\left(\Sigma_{k}^{-1}\right)+\gamma \lambda_{\min}\left(L\right),$$

and


$$\lambda_{\max}\left(H_{\mu_{k}}\right)\leq \left(N_{k}+\kappa_{0}\right)\lambda_{\max}\left(\Sigma_{k}^{-1}\right)+\gamma \lambda_{\max}\left(L\right).$$

Since the spectral condition number of a symmetric positive-definite matrix is


$$\kappa_{2}\left(H_{\mu_{k}}\right)=\frac{\lambda_{\max}\left(H_{\mu_{k}}\right)}{\lambda_{\min}\left(H_{\mu_{k}}\right)},$$

the first stated bound follows directly. For a graph Laplacian, λ_min_(*L*) = 0, which gives the reduced bound.

Finally, the covariance bounds *mI*≼Σ_*k*_≼*MI* imply $\frac{1}{M}I≼\Sigma_{k}^{-1}≼\frac{1}{m}I.$

Hence, $\lambda_{\min}\left(\Sigma_{k}^{-1}\right)\geq \frac{1}{M},\;\lambda_{\max}\left(\Sigma_{k}^{-1}\right)\leq \frac{1}{m}$.

By Gershgorin's circle theorem (Horn and Johnson, 2012), for an undirected weighted graph Laplacian,

λ_max_(*L*) ≤ 2*d*_max_.

Substituting these estimates into the reduced condition-number bound yields


$$\kappa_{2}\left(H_{\mu_{k}}\right)\leq \frac{\left(N_{k}+\kappa_{0}\right)/m+2\gamma d_{\max}}{\left(N_{k}+\kappa_{0}\right)/M}=\frac{M}{m}+\frac{2\gamma Md_{\max}}{N_{k}+\kappa_{0}}.$$

This proves the result. This bound explicitly quantifies how the covariance spectrum, graph regularization parameter, graph topology, and effective cluster size jointly affect the conditioning of the penalized mean-update system.

** Remark 5.8**. *Proposition 2 shows that the conditioning of the penalized mean update depends on the covariance spectrum, graph regularization strength, graph connectivity, and effective cluster size. Larger values of γ or *d*_max_ may increase the condition number, whereas a larger *N*_*k*_+κ_0_ reduces the relative contribution of the graph term. This bound concerns numerical conditioning and does not by itself imply an improvement or deterioration in clustering accuracy.*

** Remark 5.9** (Implication for Numerical Solution). *Under the assumptions of Proposition 2, the coefficient matrix $A_{k}=\left(\left(N_{k}+\kappa_{0}\right)\Sigma_{k}^{-1}+\gamma L\right)$ is symmetric positive definite. Consequently, the graph-regularized linear system arising in the M-step can be solved efficiently with the preconditioned conjugate gradient (PCG) method. The present study does not analyze the convergence theory of PCG; rather, the spectral condition number bound derived in Proposition 2 characterizes the linear system solved during each M-step and provides theoretical support for its numerical implementation.*

** Proposition 3** (Perturbation bound for the graph-regularized mean update). *Let $\mu_{k}^{\left(0\right)}$ denote the NIW-regularized mean estimate obtained without graph regularization (γ = 0), and let $\mu_{k}^{\left(\gamma \right)}$ denote the corresponding graph-regularized estimate for γ≥0. Define $A_{k}=\left(N_{k}+\kappa_{0}\right)\Sigma_{k}^{-1}+\gamma L$, where Σ_*k*_≻0, *L*≽0, *N*_*k*_≥0, and κ_0_>0.*

*Then*


$$\mu_{k}^{\left(\gamma \right)}-\mu_{k}^{\left(0\right)}=-\gamma A_{k}^{-1}L\mu_{k}^{\left(0\right)},$$

*and consequently*,


$$||\mu_{k}^{\left(\gamma \right)}-\mu_{k}^{\left(0\right)}|{|}_{2}\leq \frac{\gamma \lambda_{\max}\left(\Sigma_{k}\right)||L|{|}_{2}}{N_{k}+\kappa_{0}}||\mu_{k}^{\left(0\right)}|{|}_{2}.$$

*Hence, the deviation between the graph-regularized and NIW mean estimates is bounded linearly by the graph regularization parameter γ, with the bound determined by the covariance spectrum, the graph Laplacian, and the effective cluster size.*

*Proof:* Subtracting the NIW mean update from the graph-regularized update gives


$$A_{k}\left(\mu_{k}^{\left(\gamma \right)}-\mu_{k}^{\left(0\right)}\right)=-\gamma L\mu_{k}^{\left(0\right)},$$

*where* $A_{k}=\left(N_{k}+\kappa_{0}\right)\Sigma_{k}^{-1}+\gamma L$.

Since Σ_*k*_≻0, *L*≽0, and *N*_*k*_+κ_0_>0, the matrix *A*_*k*_ is symmetric positive definite. Hence,

$\mu_{k}^{\left(\gamma \right)}-\mu_{k}^{\left(0\right)}=-\gamma A_{k}^{-1}L\mu_{k}^{\left(0\right)}$.

Taking the spectral norm on both sides and using the submultiplicative property of the induced 2-norm gives


$$||\mu_{k}^{\left(\gamma \right)}-\mu_{k}^{\left(0\right)}|{|}_{2}=\gamma ||A_{k}^{-1}L\mu_{k}^{\left(0\right)}|{|}_{2}\leq \gamma ||A_{k}^{-1}|{|}_{2}||L|{|}_{2}||\mu_{k}^{\left(0\right)}|{|}_{2}.$$

Since $||A_{k}^{-1}|{|}_{2}=\frac{1}{\lambda_{\min}\left(A_{k}\right)}$, and $\lambda_{\min}\left(A_{k}\right)\geq \left(N_{k}+\kappa_{0}\right)\lambda_{\min}\left(\Sigma_{k}^{-1}\right)+\gamma \lambda_{\min}\left(L\right)$ by Weyl's inequality (Horn and Johnson, 2012), together with $\lambda_{\min}\left(\Sigma_{k}^{-1}\right)=\frac{1}{\lambda_{\max}\left(\Sigma_{k}\right)}$, the stated bound follows immediately.

** Remark 5.10**. *Proposition 3 quantifies the deviation introduced by graph regularization relative to the NIW-only mean update. The deviation increases with the regularization parameter γ and the graph spectral norm ||*L*||_2_, while it decreases as the effective cluster size *N*_*k*_+κ_0_ increases. The bound characterizes the amount of graph-induced smoothing and should not be interpreted as a guarantee of improved or degraded clustering performance.*

** Proposition 4** (Threshold characterization of the noise responsibility). *Let*


$$r_{i0}=\frac{\pi_{0}U\left(x_{i}\right)}{\overset{K}{\underset{k=1}{\sum }}\pi_{k}N\left(x_{i}|\mu_{k},\Sigma_{k}\right)+\pi_{0}U\left(x_{i}\right)}$$

*denote the posterior responsibility of the uniform noise component for observation *x*_*i*_, where π_0_>0, π_*k*_>0, *U*(*x*_*i*_)≥0, and $N\left(x_{i}|\mu_{k},\Sigma_{k}\right)\geq 0$. Then,*

1. 0 ≤ *r*_*i*0_ ≤ 1.

For any ε∈(0, 1), *r*_*i*0_ < ε if and only if


$$\pi_{0}U\left(x_{i}\right)<\frac{\varepsilon }{1-\varepsilon }\overset{K}{\underset{k=1}{\sum }}\pi_{k}N\left(x_{i}|\mu_{k},\Sigma_{k}\right).$$

For any ε∈(0, 1), *r*_*i*0_>1−ε if and only if


$$\pi_{0}U\left(x_{i}\right)>\frac{1-\varepsilon }{\varepsilon }\overset{K}{\underset{k=1}{\sum }}\pi_{k}N\left(x_{i}|\mu_{k},\Sigma_{k}\right).$$

Proof. Let $G_{i}=\overset{K}{\underset{k=1}{\sum }}\pi_{k}N\left(x_{i}|\mu_{k},\Sigma_{k}\right)$, so that $r_{i0}=\frac{\pi_{0}U\left(x_{i}\right)}{G_{i}+\pi_{0}U\left(x_{i}\right)}$.

Since *G*_*i*_≥0 and π_0_*U*(*x*_*i*_)≥0, it follows immediately that 0 ≤ *r*_*i*0_ ≤ 1, which proves Part (1).

For Part (2), substituting the above expression for *r*_*i*0_ into the inequality *r*_*i*0_ < ε and rearranging gives


$$\pi_{0}U\left(x_{i}\right)<\frac{\varepsilon }{1-\varepsilon }G_{i},$$

which, after substituting the definition of *G*_*i*_, yields the stated condition.

Similarly, substituting the expression for *r*_*i*0_ into *r*_*i*0_>1−ε and rearranging gives


$$\pi_{0}U\left(x_{i}\right)>\frac{1-\varepsilon }{\varepsilon }G_{i},$$

which proves Part (3).

** Remark 5.11**. *Proposition 4 provides explicit thresholds governing the assignment of observations to the uniform noise component. When the weighted Gaussian density dominates the weighted uniform density, the posterior noise responsibility is small, and the observation is assigned confidently to one of the Gaussian mixture components. Conversely, when the weighted uniform density dominates, the posterior responsibility approaches one, indicating confident assignment to the noise component. The parameter ε∈(0, 1) specifies the desired confidence threshold.*

The theoretical results established in this section provide several practical guarantees for the proposed MAP–EM framework. These guarantees explain how the individual components of the model contribute to numerical stability, robust parameter estimation, and reliable optimization throughout the iterative clustering process.

### 5.2 Practical implications of the theoretical results

The theoretical analysis establishes several practical implications for the proposed MAP–EM framework. The NIW prior stabilizes covariance estimation by preventing covariance degeneracy and ensuring positive-definite covariance matrices, thereby improving numerical robustness, particularly for small or overlapping clusters. Graph-Laplacian regularization yields a well-posed linear system for the mean update, while the associated condition-number analysis characterizes the computational behavior of the PCG solver and the influence of the regularization parameter γ. Furthermore, the bounded-noise responsibility ensures that observations well-explained by the Gaussian components receive negligible noise assignments, whereas atypical observations are absorbed by the uniform noise component without significantly affecting parameter estimation.

From an optimization perspective, the monotone-ascent property guarantees that the penalized objective does not decrease during the MAP–EM iterations. The local convergence result further establishes R-linear convergence in a neighborhood of a strict local maximizer, providing a theoretical basis for the observed convergence behavior. Finally, the split–merge refinement terminates after a finite number of accepted moves because only objective-improving structural modifications are permitted. Taken together, these results demonstrate that the proposed MAP–EM framework is mathematically well-posed, numerically stable, and well-suited for clustering high-dimensional, noisy, and structurally complex document collections.

## 6 Experimental analysis

All experiments were conducted on a Dell Inspiron laptop equipped with an 11th Gen Intel Core i7 processor, 16 GB RAM, and a 64-bit operating system. The proposed MAP–EM model was evaluated on synthetic datasets for ablation, scalability, and sensitivity studies. The benchmark datasets were used for the document clustering task. The proposed MAP–EM model involves the hyperparameters γ, β_noise_, and β, together with the algorithmic settings *k*_*nn*_ and max_moves. Here, γ denotes the graph regularization strength, β_noise_ denotes the contribution of the uniform noise component, and β denotes the complexity-penalty parameter used during model refinement. The quantities *k*_*NN*_ and max_moves control graph construction and refinement behavior, respectively.

The clustering performance of the proposed MAP–EM model was compared with the performance of classical methods such as *k*-means, spectral clustering, expectation–maximization Gaussian mixture model (EM–GMM), variational Bayesian Gaussian mixture model (VB–GMM), and Laplacian regularized Gaussian mixture model (LapGMM). Clustering performance was assessed using external and internal validation measures (Halkidi et al., 2001). Adjusted Rand Index (ARI), Normalized Mutual Information (NMI), F1 score, accuracy, and purity were used as external measures based on available ground-truth labels. Silhouette score and Dunn index were used as internal measures to evaluate cluster compactness and separation. For all reported measures, higher values indicate better clustering performance.

### 6.1 Ablation study

To assess the contribution of each component of the proposed MAP–EM model, an extensive ablation study was performed. Each configuration was designed by isolating a single module or by excluding a single module while retaining the others. The modules considered were the normal–inverse–Wishart (NIW) priors, graph regularization, noise component, and split–merge adaptation. All experiments were executed for 15 independent runs across multiple data sets to ensure the performance and statistical reliability of the results.

#### 6.1.1 Synthetic dataset

The ablation study was conducted on five synthetic datasets, each designed to represent a distinct clustering challenge under controlled conditions. All datasets consist of continuous-valued numerical attributes and were generated with known ground-truth cluster labels. These labels were used to assess clustering quality using external validation indices, and cluster-count error was evaluated directly by comparing the estimated number of clusters with the true number. This includes a well-separated dataset with 300 samples, 2 features, and 3 clearly separated clusters; an overlapping dataset with 300 samples, 2 features, and 3 clusters with increased dispersion; a with outliers dataset with 300 samples, 2 features, and 3 main clusters together with 20 uniformly distributed outlier points; a non-spherical dataset with 300 samples and 2 features arranged in a curved two-cluster structure; and a high-dimensional dataset with 200 samples, 10 features and 4 clusters. Together, these datasets were chosen to evaluate the proposed model across different clustering conditions, including well-separated, overlapping, outlier-contaminated, non-spherical, and high-dimensional settings. All experiments were repeated over multiple independent runs using controlled random seeds to ensure reproducibility. The characteristics of the synthetic datasets used in the ablation study are summarized in Table 2.

**Table 2:** Characteristics of the synthetic datasets used in the ablation study, including sample size, feature dimension, number of clusters, and clustering characteristics.

Dataset	Samples	Features	True clusters	Attribute type	Data characteristic
Well-separated	300	2	3	Continuous	Clearly separated cluster groups
Overlapping	300	2	3	Continuous	Increased dispersion with substantial cluster overlap
With outliers	300	2	3	Continuous	Three clusters with 20 added anomalous points
Non-spherical	300	2	2	Continuous	Curved, non-Gaussian cluster structure
High-dimensional	200	10	4	Continuous	Higher feature dimension than the other synthetic settings

#### 6.1.2 Performance analysis

The ablation study shows that the tested configurations differ noticeably in their clustering performance. The highest ARI was obtained by the only NIW priors configuration (0.7143 ± 0.2411), followed by only split–merge (0.7047 ± 0.2510). The all except split–merge configuration also achieved a higher ARI (0.6682 ± 0.2721) than the proposed MAP–EM model. The proposed MAP–EM model obtained 0.5671 ± 0.2367, which was the lowest among the tested configurations.

**Figure 2:**
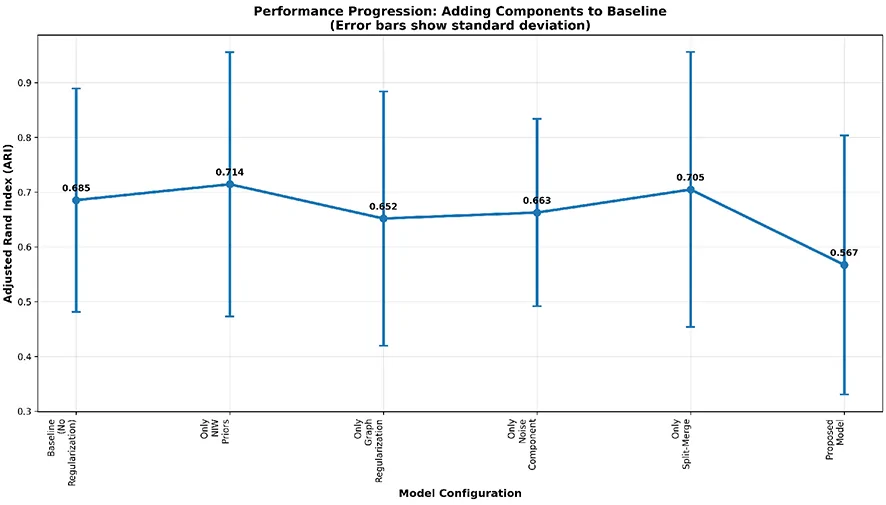
Performance progression across incremental model configurations, showing the mean ARI for the baseline, single-component configurations, and the proposed MAP–EM model.

These results show that the proposed MAP–EM model does not provide the strongest clustering agreement in terms of the ARI on the synthetic ablation setting. The ablation results also indicate that the effects of the individual components are not strictly additive. In particular, although the split–merge module performs strongly in isolation, the proposed MAP–EM model does not improve ARI when this module is combined with all the other components. This suggests that the contribution of split–merge is configuration-dependent and interacts with the other regularization components rather than yielding a uniformly beneficial improvement in ARI. In terms of cluster-count estimation, the proposed MAP–EM model shows a low mean absolute cluster-count error, though this advantage is not exclusive to it. Figure 2 illustrates how clustering performance changes across configurations and, in particular, shows the higher ARI values achieved by the NIW-only and split–merge-only variants relative to the full proposed MAP–EM model. Table 3 reports the mean ARI, the standard deviation of ARI, and the mean absolute cluster-count error |*K*_true_−*K*_found_| for all configurations.

**Table 3:** Ablation results of the unified MAP–EM model across configurations, showing mean ARI, standard deviation of ARI, and mean absolute cluster-count error.

Configuration	ARI mean	**ARI Std**.	**Mean |*K*_true_−*K*_found_|**
Only NIW priors	0.7143	0.2411	2.0
Only split–merge	0.7047	0.2510	7.8
All except graph	0.6861	0.2734	1.0
Baseline (no regularization)	0.6853	0.2040	2.0
All except split–merge	0.6682	0.2721	2.0
Only noise component	0.6626	0.1711	2.0
Only graph regularization	0.6518	0.2320	2.0
All Except NIW	0.6244	0.2999	12.6
All except noise	0.5675	0.2363	1.0
Proposed MAP–EM model	0.5671	0.2367	1.2

#### 6.1.3 Performance across dataset types

On the well-separated dataset, all configurations achieved perfect clustering performance (ARI = 1.000), indicating that this setting does not differentiate among the configurations. On the overlapping dataset, the strongest result was obtained by only split–merge (0.610), followed by only NIW priors (0.525), suggesting that these components are more effective in dealing with assignment ambiguity. In contrast, the non-spherical dataset remained challenging for all configurations, with ARI values ranging only from 0.288 to 0.437, indicating limited clustering quality in this setting. With the outliers dataset, the results show a strong dependence on noise handling. That is, the configurations that retain the noise component achieved ARI values as high as 0.979, whereas removing the noise component substantially reduced the ARI to 0.568. This shows that performance with outlier datasets depends strongly on the inclusion of the noise component and is not uniform across configurations. On the high-dimensional dataset, the ARI values were moderate, ranging from 0.439 to 0.631, which suggests only limited separation among the configurations in this setting. The proposed MAP–EM model and the all-except-noise configuration also produced very similar ARI values on several dataset types, suggesting that the effect of the noise component on ARI is limited in these cases. Table 4 reports the absolute ARI values for the selected configurations, including the baseline and the proposed MAP–EM model, across the five synthetic data sets.

**Table 4:** ARI values of the selected configurations across the five synthetic dataset types.

Dataset type	Baseline	All except graph	All except NIW	All except noise	Only NIW priors	Only split–merge	Proposed MAP–EM
High-dimensional	0.564	0.631	0.450	0.439	0.631	0.575	0.439
Non-spherical	0.452	0.341	0.288	0.343	0.437	0.371	0.341
Overlapping	0576	0.480	0.441	0.488	0.525	0.610	0.488
Well-separated	0.867	1.000	1.000	1.000	1.000	1.000	1.000
With outliers	0.968	0.979	0.943	0.568	0.979	0.968	0.568

#### 6.1.4 Cluster count selection accuracy

**Figure 3:**
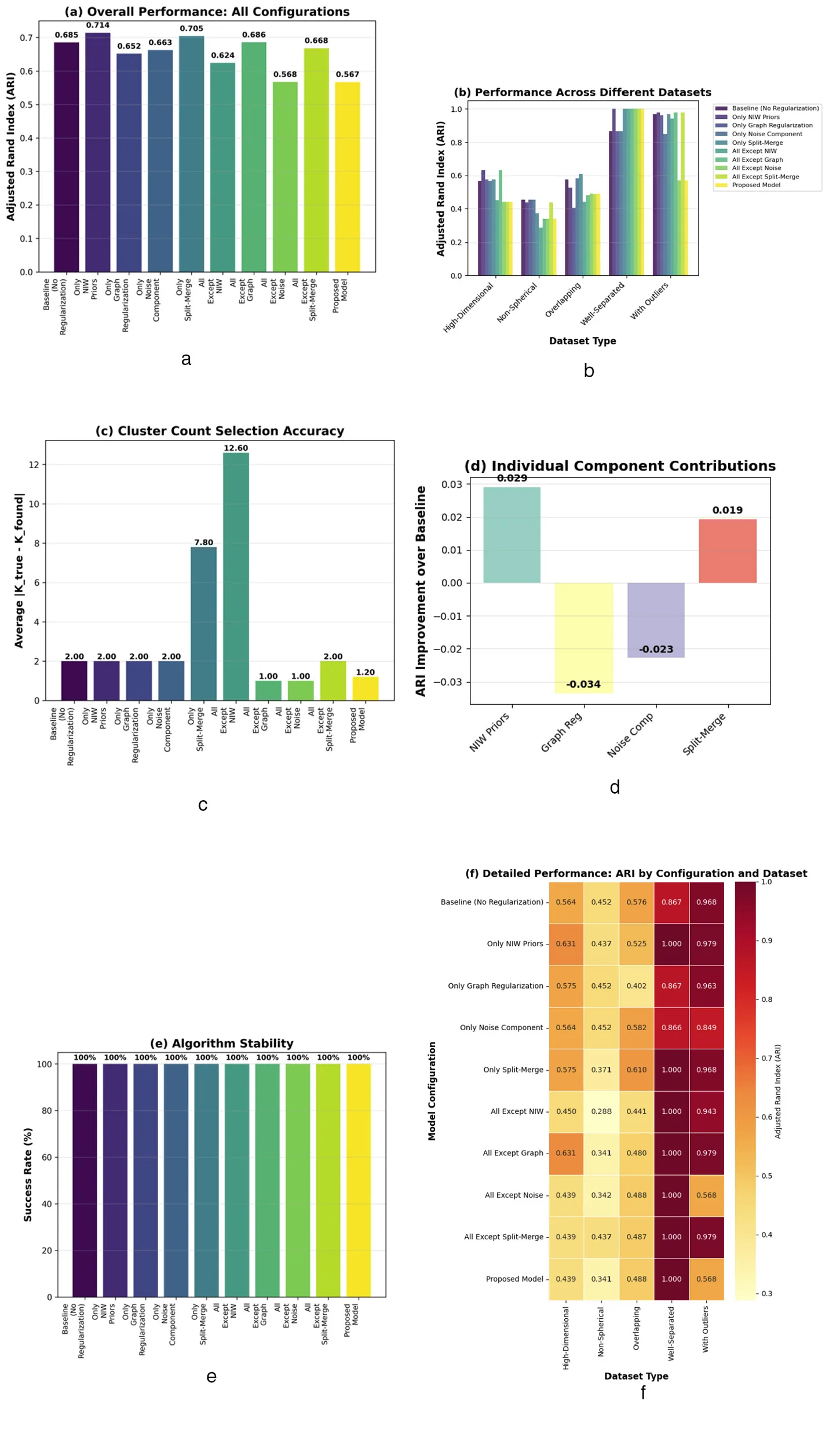
Comprehensive ablation study of the proposed MAP–EM model. **(a)** Mean ARI of selected configurations. **(b)** Mean ARI across different synthetic datasets for selected configuration. **(c)** Mean absolute cluster-count error |*K*_true_−*K*_found_| across configurations. **(d)** Change in mean ARI relative to the baseline for individual components. **(e)** Stability rate across ablation configurations. **(f)** Heatmap of ARI values across selected configurations and synthetic datasets.

The ablation results also show noticeable differences in the ability of the tested configurations to recover the correct number of clusters. Figure 3c shows that the proposed MAP–EM model achieves a low cluster-count error, with a mean absolute deviation of 1.2 clusters. At the same time, several other configurations also achieve similarly low errors, particularly all except graph and all except noise, both with an error of 1.0. By contrast, much larger deviations are observed only for split–merge (7.8) and all except NIW (12.6). Therefore, the low cluster-count error of the proposed MAP–EM model cannot be attributed to the split–merge step alone. Rather, it shows that multiple integrated configurations help to achieve a comparable cluster-count accuracy. Table 3 reports the mean absolute cluster-count estimation errors |*K*_true_−*K*_found_| for each configuration.

#### 6.1.5 Trade-off between ARI and cluster-count

The ablation results further highlight a practical trade-off between clustering agreement and accurate recovery of the number of clusters. The proposed MAP–EM model consistently shows lower cluster-count error than the baseline and NIW-only configurations, indicating more accurate estimation of the number of clusters. This is practically important because an incorrect number of clusters can lead to over-segmentation or under-segmentation of the data and can reduce the interpretability of the recovered structure. From this perspective, a moderate reduction in ARI may be acceptable when improved model selection is itself a primary objective.

At the same time, the results show that this trade-off is dataset dependent. The proposed MAP–EM matches the best performance on the well-separated dataset (ARI = 1.000), but yields lower ARI on the high-dimensional, non-spherical, overlapping, and with outliers datasets. In particular, the loss is substantial in the with outliers case, where the proposed model attains ARI = 0.568, compared with 0.968 for the baseline and 0.979 for the NIW only variant. Therefore, the practical implication is not that lower ARI is always acceptable, but rather that improved estimation of the number of clusters may justify some loss in ARI when model-order accuracy is crucial for interpretation. The proposed MAP–EM model is most attractive in applications where reliable recovery of the number of clusters is a priority, whereas the baseline or NIW only configurations may be preferable when achieving higher ARI is the primary objective.

The model stability was 1.000 throughout the experiments, indicating that all runs converged successfully without instability. Therefore, the observed trade-off arises between ARI and cluster-count error rather than from variation in convergence stability.

The ARI–cluster-count trade-off should be interpreted with care. A higher ARI is desirable when the main goal is to match known ground-truth labels as closely as possible. In such cases, a method that gives stronger ARI may be preferable even if it does not recover the exact number of clusters. Therefore, cluster-count accuracy should not be treated as universally more important than ARI.

Accurate cluster-count estimation is useful in exploratory settings where the number of groups is not known in advance. This situation is common in document organization, topic discovery, customer feedback analysis, and knowledge discovery from large text collections. In these applications, overestimating the number of clusters can split one meaningful topic into several small groups, while underestimating the number of clusters can merge distinct topics and reduce interpretability. Hence, a moderate reduction in ARI may be acceptable when the main objective is to obtain a stable and interpretable grouping structure with a reasonable estimate of the number of latent groups.

**Figure 4:**
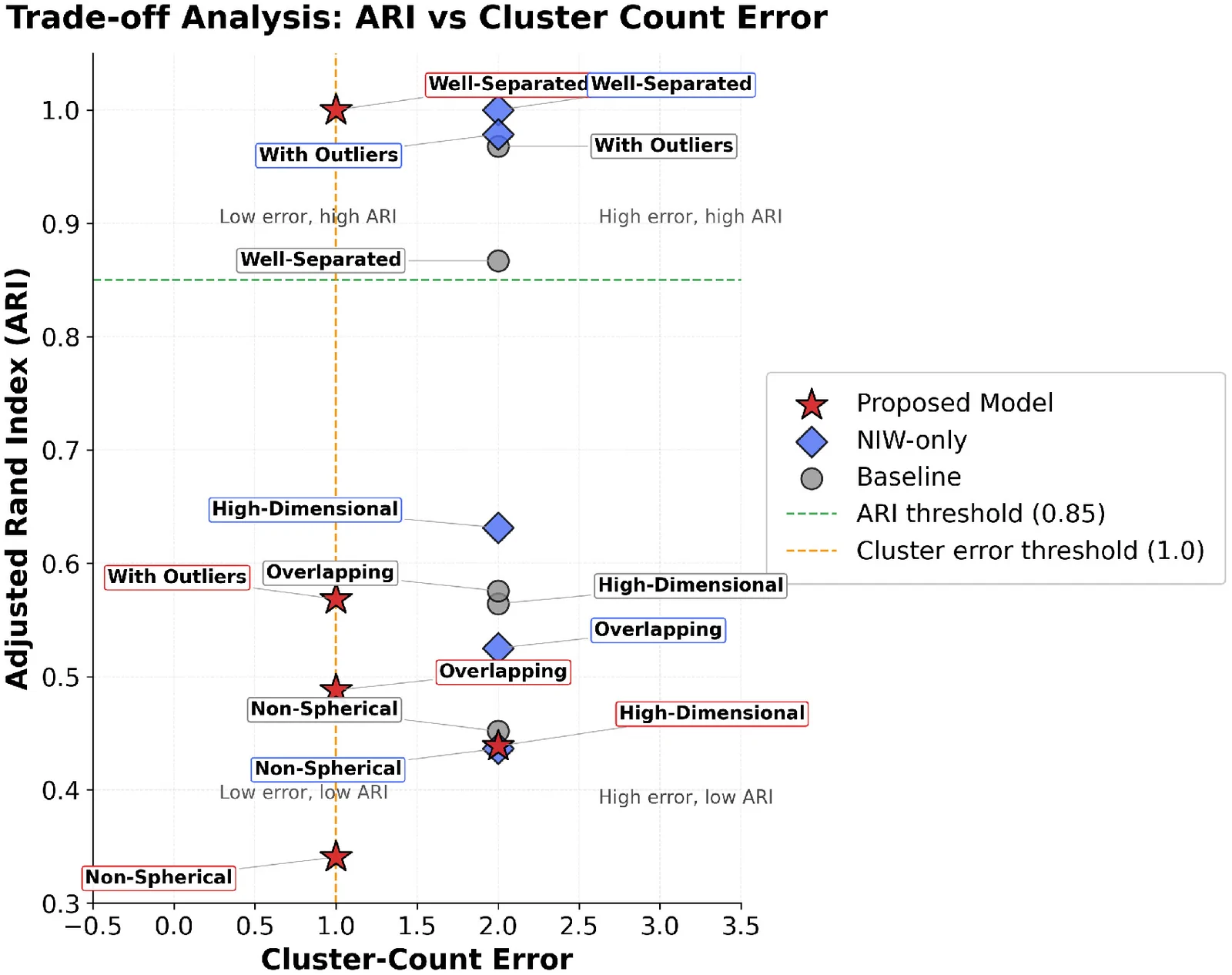
Visual representation of trade-off between clustering agreement, measured by ARI, and cluster-count error across synthetic datasets and selected model configurations.

Figure 4 reveals a clear trade-off between clustering agreement and cluster-count recovery across synthetic dataset types, shown in terms of ARI and cluster-count error. Lower cluster-count error and higher ARI are more desirable. The dashed lines indicate the reference thresholds for ARI and cluster-count error, helping to distinguish favorable and unfavorable regions of performance.

#### 6.1.6 Component effectiveness analysis

To understand how each module contributes to the MAP–EM approach, the marginal effect of each component was compared with the baseline configuration. The NIW priors and split–merge mechanism showed positive contributions to ARI, with increases of (+0.029) and (+0.019), respectively. Conversely, graph regularization (-0.034) and the noise component (–0.023) produced lower ARI values when used in isolation.

The results also show that the effects of the components are not strictly additive. Although NIW priors and graph regularization each influence the model separately, their combined configuration achieved an ARI of 0.6702, which is higher than graph regularization alone but lower than NIW priors alone. This suggests that combining useful components does not always lead to a stronger improvement, since the modules may interact with each other in different ways depending on the data structure.

Table 5 summarizes the marginal contribution of each component as the ARI difference relative to the baseline. Figure 3d shows the corresponding visual comparison of marginal ARI changes.

**Table 5:** Change in ARI relative to the baseline for individual components and the NIW + graph regularization combination.

Configuration	ARI	ARI difference from baseline
Baseline (no regularization)	0.6853	0.0000
Only NIW priors	0.7143	+0.0290
Only graph regularization	0.6518	–0.0335
Only noise component	0.6626	–0.0227
Only Split–Merge	0.7047	+0.0194
NIW + graph regularization	0.6702	–0.0151

#### 6.1.7 Algorithm stability

All the model configurations achieved 100% convergence stability, indicating stable behavior across the runs. This outcome is depicted in Figure 3e. No covariance singularities or numerical instabilities were observed, in contrast to the difficulties that can arise in classical EM frameworks under small-sample or collinear conditions.

#### 6.1.8 Joint ARI comparison across configurations and dataset types

To further examine how clustering agreement varies across dataset types, Figure 3f presents a configuration-wise heatmap of ARI values. The heatmap shows that clustering performance depends strongly on both the dataset type and the selected model components, with higher ARI often achieved by single-component or less-constrained configurations rather than by the proposed MAP–EM model.

From the ablation study, the following observations were made.

1. NIW priors provide the strongest positive individual contribution to ARI among the single-component settings.
2. Split–merge can improve performance in some configurations, but its effect is not uniformly positive when combined with other modules.
3. Graph regularization and the noise component do not improve ARI when applied in isolation.
4. Across the tested configurations, the MAP–EM framework shows stable convergence across runs, whereas the clustering performance varied considerably across the dataset types.

The ablation study shows that the contribution of each component depends strongly on the configuration in which it is used. Rather than indicating a uniformly additive effect, the results suggest that combining multiple regularization and adaptation mechanisms introduces trade-offs mainly between clustering agreement and cluster-count estimation, whereas convergence stability remained high across all the configurations. Figure 3 summarizes these results.

The ablation results show that the proposed MAP–EM model does not always achieve the highest ARI when compared with simpler configurations of the method. In several cases, configurations using only the NIW prior or only the split–merge mechanism perform comparably to or better than in terms of ARI. This indicates that adding all components together does not guarantee better clustering agreement across all datasets. The role of the proposed model is to handle settings in which several clustering challenges co-occur, including noise, overlap, an uncertain number of clusters, and feature-level structural relationships.

The proposed MAP–EM model is more suitable when the data contain multiple sources of difficulty simultaneously. These include noisy observations, overlapping groups, uncertain topic structure, and meaningful relationships among features that can be represented using a connected feature graph. Such settings often arise in exploratory document clustering and topic discovery, where the number of latent groups is unknown in advance, and the documents may contain mixed or weakly separated themes. In such cases, the combined use of NIW prior regularization, graph-based smoothing, noise modeling, and split–merge refinement gives a broader clustering model than any single reduced variant.

The proposed model may not be necessary when the data are relatively clean or well-separated. For example, if the major issue is covariance stabilization or parameter regularization, the NIW-only variant may be sufficient. If the main issue is cluster-number adjustment, the split–merge-only variant may perform competitively. At the same time, the results indicate that the added constraints do not compromise convergence stability. The findings are best interpreted as reflecting trade-offs between evaluation criteria, not as uniformly favorable gains in performance. Thus, the proposed MAP–EM model should be viewed as better suited to mixed and moderately complex clustering settings, not as a universally superior option for every dataset.

### 6.2 Sensitivity analysis of feature-graph construction and graph regularization

To examine the effect of the graph component in a controlled setting, an additional sensitivity analysis was conducted using a synthetic dataset rather than the benchmark document corpora used in the main experimental comparison. The synthetic dataset consisted of 500 samples with 10 features and 5 true clusters, generated as well-separated Gaussian blobs with approximately 5% label noise. This controlled setting was used only to assess the behavior of the graph-related hyperparameters, not as a replacement for real-data evaluation.

Since the proposed MAP–EM model incorporates structural information through the Laplacian penalty


$$\frac{\gamma }{2}\overset{K}{\underset{k=1}{\sum }}\mu_{k}^{⊤}L\mu_{k},$$

the sensitivity analysis was designed to examine both the construction of the feature graph and the strength of the graph regularization. The neighborhood size of the *k*-nearest-neighbor feature graph is denoted by *k*_NN_ to distinguish it from the number of mixture components. In this study, the graph neighborhood size was varied from *k*_NN_ = 2 to *k*_NN_ = 9, while the graph regularization weight γ was varied over the range {0.001, 0.01, 0.1, 0.5, 1, 2, 5}, with the remaining parameters kept fixed.

The results show that the effect of the graph term depends mainly on the value of γ. For very small values of γ, the clustering metrics remain almost unchanged across neighborhood sizes, suggesting that the graph penalty is too weak to modify the MAP–EM solution in a noticeable way. For moderate values such as γ = 1 and γ = 2, ARI increases slightly and remains comparatively stable across the tested graph constructions. The mean ARI rises from 0.6770 to 0.6836, keeping the corresponding standard deviations small. The NMI values follow the same general pattern. When γ is increased to 5, the highest single ARI and NMI are obtained at *k*_NN_ = 3, but the summary statistics indicate much larger variability across graph settings. In particular, the ARI standard deviation at γ = 5 is 0.3177, which is substantially higher than at moderate settings. This suggests that strong graph regularization makes clustering performance more sensitive to the choice of graph construction parameter *k*_NN_.

**Figure 5:**
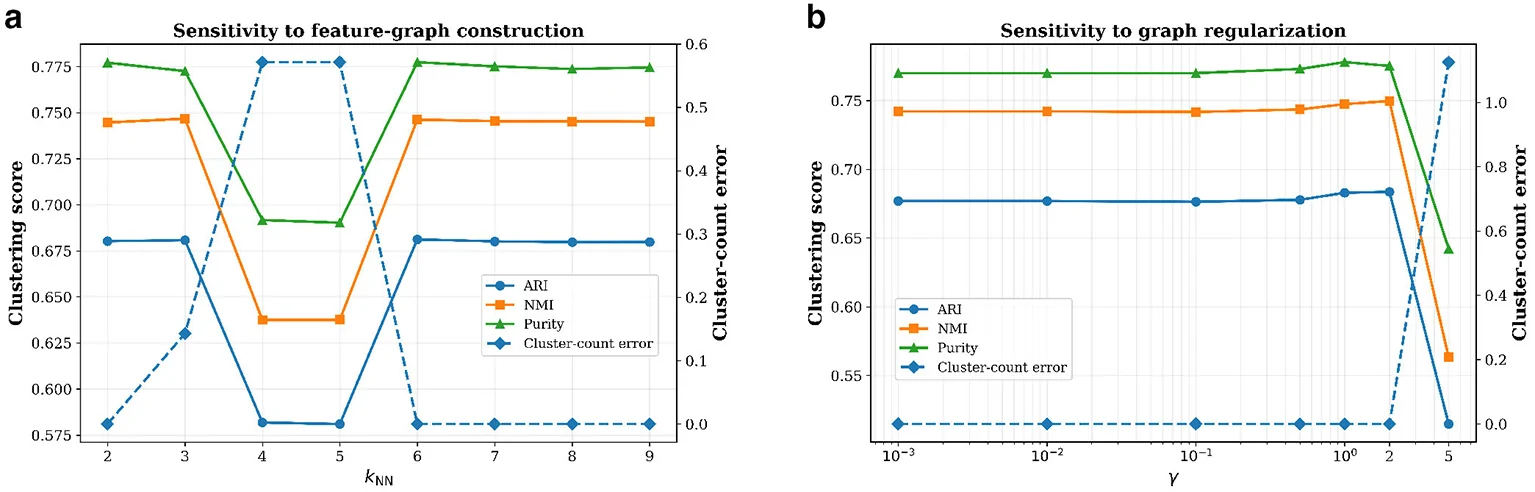
Sensitivity analysis of the graph-based component of the proposed MAP–EM model. The left panel shows the effect of varying the feature-graph construction parameter *k*_NN_, while the right panel shows the effect of varying the graph regularization weight γ. **(a)** Sensitivity to feature-graph construction with varying *k*_NN_. **(b)** Sensitivity to graph regularization with varying γ.

Table 6 reports representative configurations, including the best single-scoring case, while Table 7 summarizes the behavior across graph neighborhood sizes. Table 8 gives examples of unstable behavior under very strong graph regularization. Since the true number of clusters is *K*_true_ = 5, these failed cases correspond to a cluster-count error of 4, indicating a collapse to a single recovered cluster. These results indicate that the effect of the graph component depends on the choice of parameter. Under the tested settings, moderate graph regularization is associated with more stable behavior than the stronger setting γ = 5, even though the latter attains the highest ARI and NMI in one configuration. The results also suggest that, within the tested range, variation in clustering performance is more pronounced across γ than across *k*_NN_. Figure 5 presents the sensitivity of the graph-based component with respect to the feature-graph construction parameter *k*_NN_ and the graph regularization weight γ.

**Table 6:** Sensitivity analysis of the proposed MAP–EM model with respect to the feature-graph neighborhood size *k*_NN_ and the graph regularization weight γ.

*k*_NN_	γ	ARI	NMI	Purity	Silhouette	Dunn
3	0.001	0.6770	0.7422	0.7700	0.2506	0.1228
2	1.000	0.6837	0.7465	0.7880	0.2416	0.1349
3	1.000	0.6805	0.7429	0.7780	0.2477	0.1349
6	1.000	0.6869	0.7526	0.7800	0.2521	0.1377
3	2.000	0.6869	0.7526	0.7800	0.2521	0.1377
6	2.000	0.6848	0.7507	0.7760	0.2613	0.1566
3	5.000	0.6924	0.7647	0.7700	0.3027	0.1566

**Table 7:** Summary statistics of clustering performance across graph neighborhood sizes for each graph regularization weight γ, computed over *k*_NN_ = 2, …, 9.

γ	Mean ARI	Std. ARI	Max ARI	Mean cluster error	Std. cluster error
0.001	0.6770	0.0000	0.6770	0.000	0.0000
0.010	0.6770	0.0000	0.6770	0.000	0.0000
0.100	0.6764	0.0010	0.6770	0.000	0.0000
0.500	0.6778	0.0037	0.6812	0.000	0.0000
1.000	0.6829	0.0021	0.6869	0.000	0.0000
2.000	0.6836	0.0024	0.6869	0.000	0.0000
5.000	0.5147	0.3177	0.6924	1.125	1.8077

**Table 8:** Examples of unstable clustering behavior under very strong graph regularization, illustrating failure cases such as collapse to an incorrect number of clusters.

*k*_NN_	γ	ARI	NMI	Purity	Silhouette	Dunn	Cluster error
4	5.000	0.0000	0.0000	0.2040	0.0000	0.0000	4
5	5.000	0.0000	0.0000	0.2040	0.0000	0.0000	4

### 6.3 Sensitivity analysis of the proposed MAP–EM model

Sensitivity analysis was conducted on a challenging synthetic dataset comprising 1,152 samples, 25 features, and 7 underlying clusters, along with overlapping groups, anisotropic covariance structures, noisy features, and outliers. The results reveal a clear trade-off between clustering quality and cluster count error. Moderate values of γ, β_noise_, and *k*_*NN*_ improve ARI, NMI, and purity, whereas larger values increase the cluster-count error, indicating progressive cluster merging. Similarly, increasing β reduces clustering quality and worsens model-order recovery, while increasing max_moves yields only marginal improvements in external clustering scores but steadily increases the cluster-count error. Overall, these results suggest that balanced intermediate settings are preferable to hyperparameter choices that optimize only a single performance measure.

Table 9 summarizes these findings by reporting, for each hyperparameter, the tested range, the region that yields the strongest external clustering scores, and the balanced region that better preserves both clustering quality and cluster-count recovery. Taken together, the table indicates that the score-maximizing region is not always the most practically desirable, as it may coincide with a substantial increase in cluster-count error.

**Table 9:** Sensitivity trends of the proposed MAP–EM model on a synthetic dataset, showing the tested range, best-score region, and balanced region for each hyperparameter.

Hyperparameter	Tested range	Best-score region	Balanced region
γ	0, 0.001, 0.01, 0.05, 0.1, 0.25, 0.5, 1, 2, 3, 5	0.5–1.0	0.1
β_noise_	0, 0.01, 0.03, 0.05, 0.08, 0.1, 0.15, 0.2	0.05–0.2	0.03
β	0.1, 0.25, 0.5, 1, 2, 3, 5, 10	0.1	0.1–0.25
*k*_*NN*_	3, 5, 8, 10, 12, 15, 20, 25, 30	8–10	3
max_moves	0, 1, 2, 4, 6, 8, 10, 12	6–12	0–1

#### 6.3.1 Effect of γ

The performance curves for γ show that ARI, NMI, and purity improve from very small values up to approximately γ = 0.5–1.0, and then gradually decline for larger values. The cluster-count error remains 0 up to about γ = 0.1, increases to around 0.6 at γ = 0.25, and reaches 1.0 for γ≥0.5. This suggests that moderate graph regularization is beneficial, whereas stronger regularization leads to over-smoothing and underestimation of the true number of clusters.

#### 6.3.2 Effect of β_noise_

The β_noise_ curves show a small but clear improvement in ARI, NMI, and purity as the value increases from 0 to about 0.03–0.05, after which performance largely plateaus. The cluster-count error remains 0 up to β_noise_ = 0.03 but jumps to 1.0 from β_noise_ = 0.05 onward. Thus, a small noise contribution is beneficial, whereas excessive noise weight begins to absorb genuine cluster structure.

#### 6.3.3 Effect of β

For β, the best external scores are obtained at smaller values, particularly around 0.1–0.5, and then decline as the penalty increases. At the same time, the cluster-count error rises from about 0.4 at β = 0.1 to 1.0 for β≥1. Hence, stronger prior shrinkage reduces clustering quality and worsens model-order recovery.

#### 6.3.4 Effect of *k*_*NN*_

For *k*_*NN*_, ARI, NMI, and purity improve slightly from *k*_*NN*_ = 3 to about *k*_*NN*_ = 8–15, after which the curves level off or decline marginally. By contrast, the cluster-count error increases sharply, from 0 at *k*_*NN*_ = 3 to 0.6 at *k*_*NN*_ = 5, 0.8 at *k*_*NN*_ = 8, and 1.0 for *k*_*NN*_≥10. Thus, denser graph construction provides only modest gains in external scores while also promoting cluster merging.

#### 6.3.5 Effect of max_moves

The performance curves for max_moves remain nearly flat, with only a slight increase in ARI, NMI, and purity as max_moves increases. Conversely, the cluster count error rises steadily from about 0.2 at max_moves = 0 to 1.0 for max_moves≥6. This suggests that additional refinement moves provide only limited benefit in clustering quality while increasingly distorting the recovered number of clusters.

**Figure 6:**
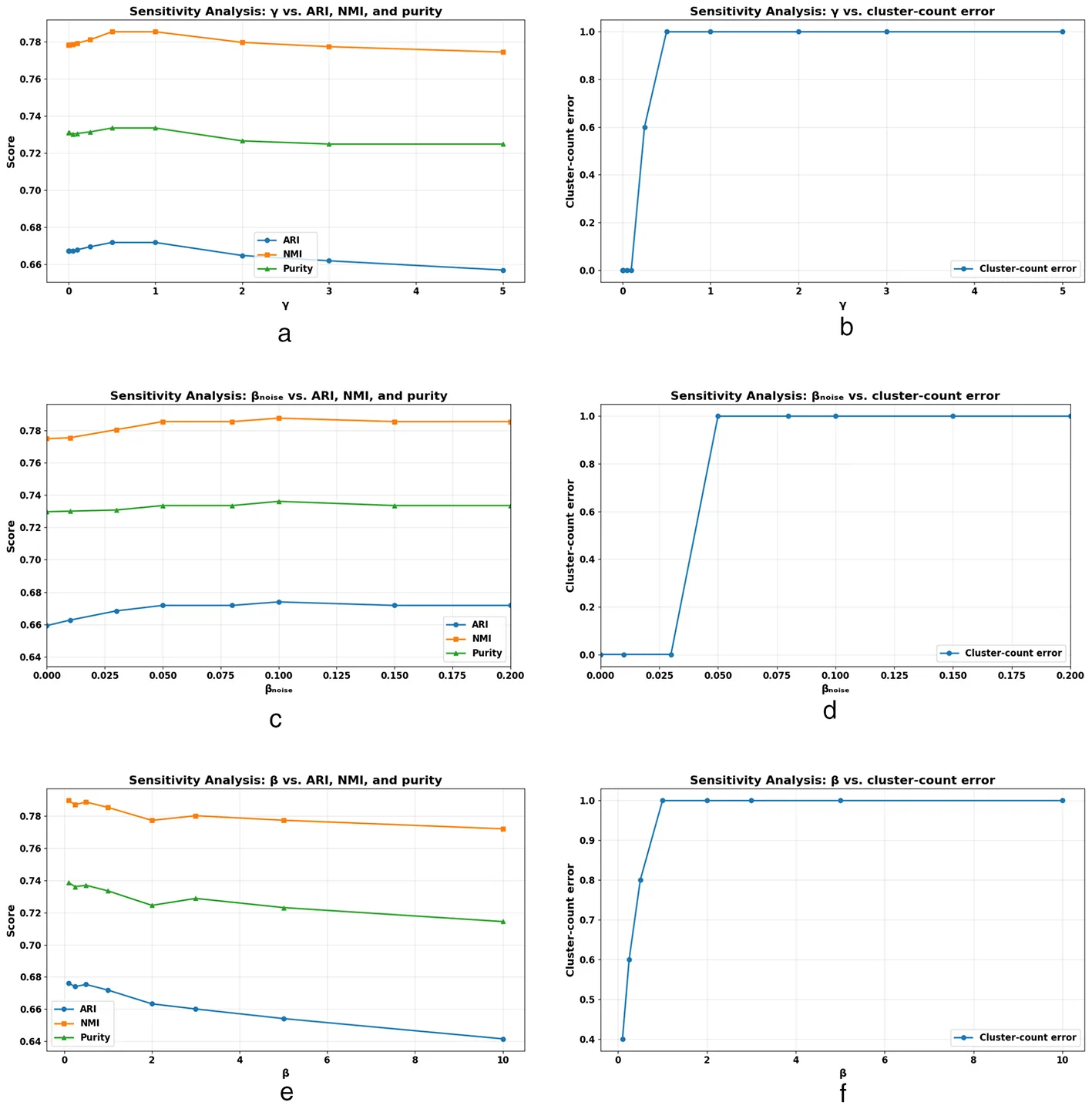
Sensitivity analysis of the proposed MAP–EM model with respect to the graph regularization weight γ, the noise-component weight β_noise_, and the complexity-penalty parameter β. For each hyperparameter, the left panel shows the variation in ARI, NMI, and purity, while the right panel shows the corresponding cluster-count error. **(a)** γ: ARI, NMI, and purity. **(b)** γ: cluster-count error. **(c)** β_noise_: ARI, NMI, and purity. **(d)** β_noise_: cluster-count error. **(e)** β: ARI, NMI, and purity. **(f)** β: cluster-count error.

These trends are visually summarized in Figure 6. The left panels show how ARI, NMI, and purity vary with γ, β_noise_, and β, whereas the right panels show the corresponding cluster-count error. These plots provide a clear visual summary that moderate parameter values yield the most balanced behavior, while stronger regularization or noise weighting tends to increase cluster merging and degrade model-order recovery.

**Figure 7:**
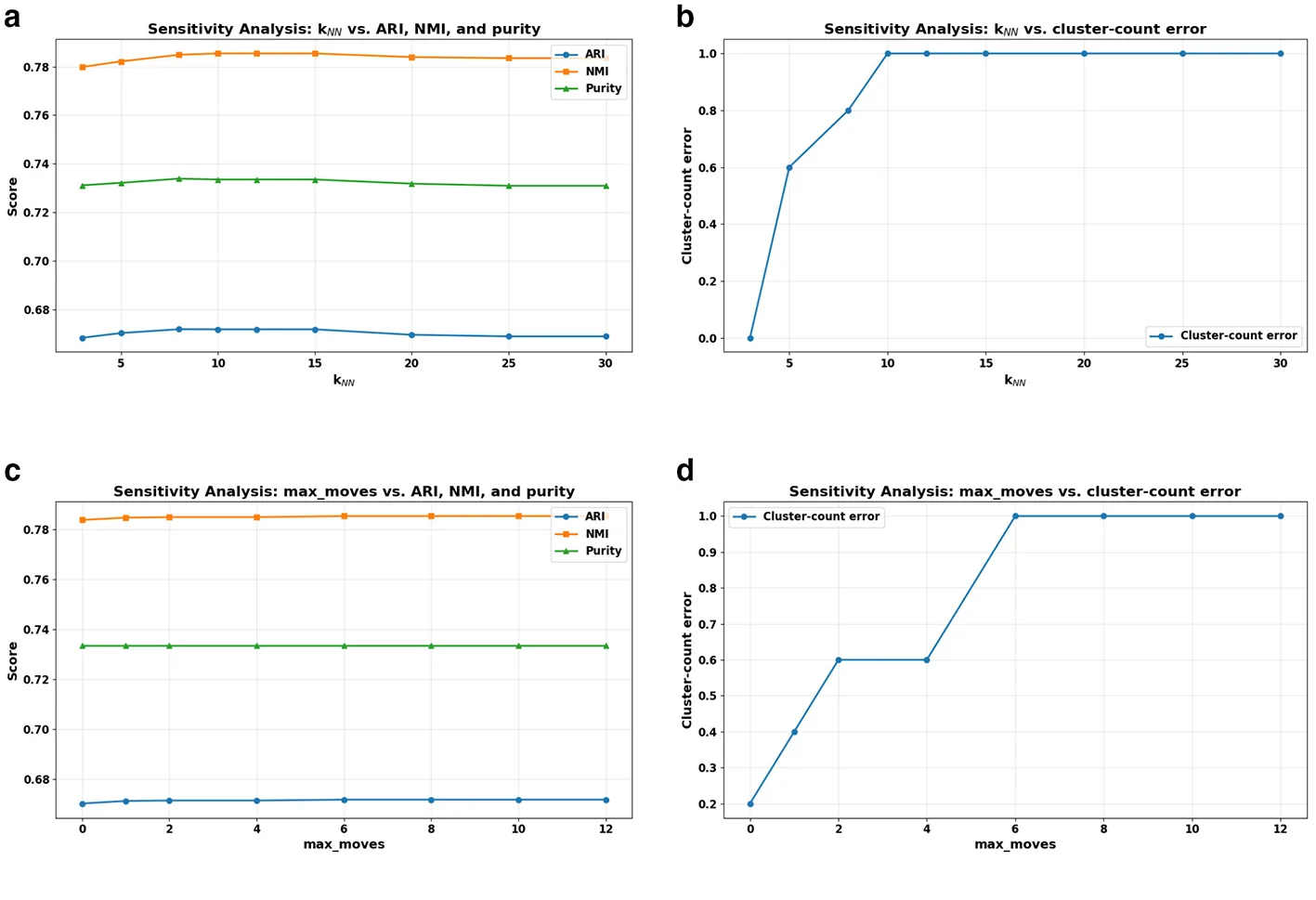
Sensitivity analysis of the proposed MAP–EM model with respect to the feature-graph neighborhood size *k*_NN_ and the maximum number of split–merge refinement moves. The left panel shows ARI, NMI, and purity, while the right panel shows the corresponding cluster-count error. **(a)** *k*_NN_: ARI, NMI, and purity. **(b)** *k*_NN_: cluster-count error. **(c)** max_moves: ARI, NMI, and purity. **(d)** max_moves: cluster-count error.

A similar pattern appears in Figure 7, which summarizes the effects of *k*_*NN*_ and max_moves. In both cases, modest increases can produce slight improvements in external clustering measures, but larger values steadily increase the cluster-count error, indicating that any apparent gain in clustering score is accompanied by deterioration in the recovered number of clusters.

### 6.4 Scalability and runtime analysis

To examine scalability under controlled conditions, synthetic datasets were generated in a fixed 10-dimensional feature space. Each dataset consisted of isotropic Gaussian clusters with spherical covariance structure and a common cluster spread of 1.2. The experiments varied either the sample size *N* or the number of clusters *K* while keeping the remaining generation settings fixed. The dataset size was varied from *N* = 100 to *N* = 2000, and the number of clusters was varied from *K* = 2 to *K* = 500. All synthetic datasets were generated under fixed settings for reproducibility. Specifically, the dataset-size experiment was fixed for *K* = 12, whereas the cluster-scaling experiment was fixed for *N* = 1750.

Scalability was examined by varying the dataset size and the number of clusters on these synthetic datasets. EM–GMM consistently achieved the lowest runtime due to its simpler update structure. The proposed MAP–EM method required additional computational time due to the NIW prior, graph regularization, noise modeling, and split–merge adaptation. VB–GMM exhibited intermediate runtime behavior in the dataset-size experiment, while both VB–GMM and the proposed MAP–EM showed increased computational cost as the number of clusters grew.

**Figure 8:**
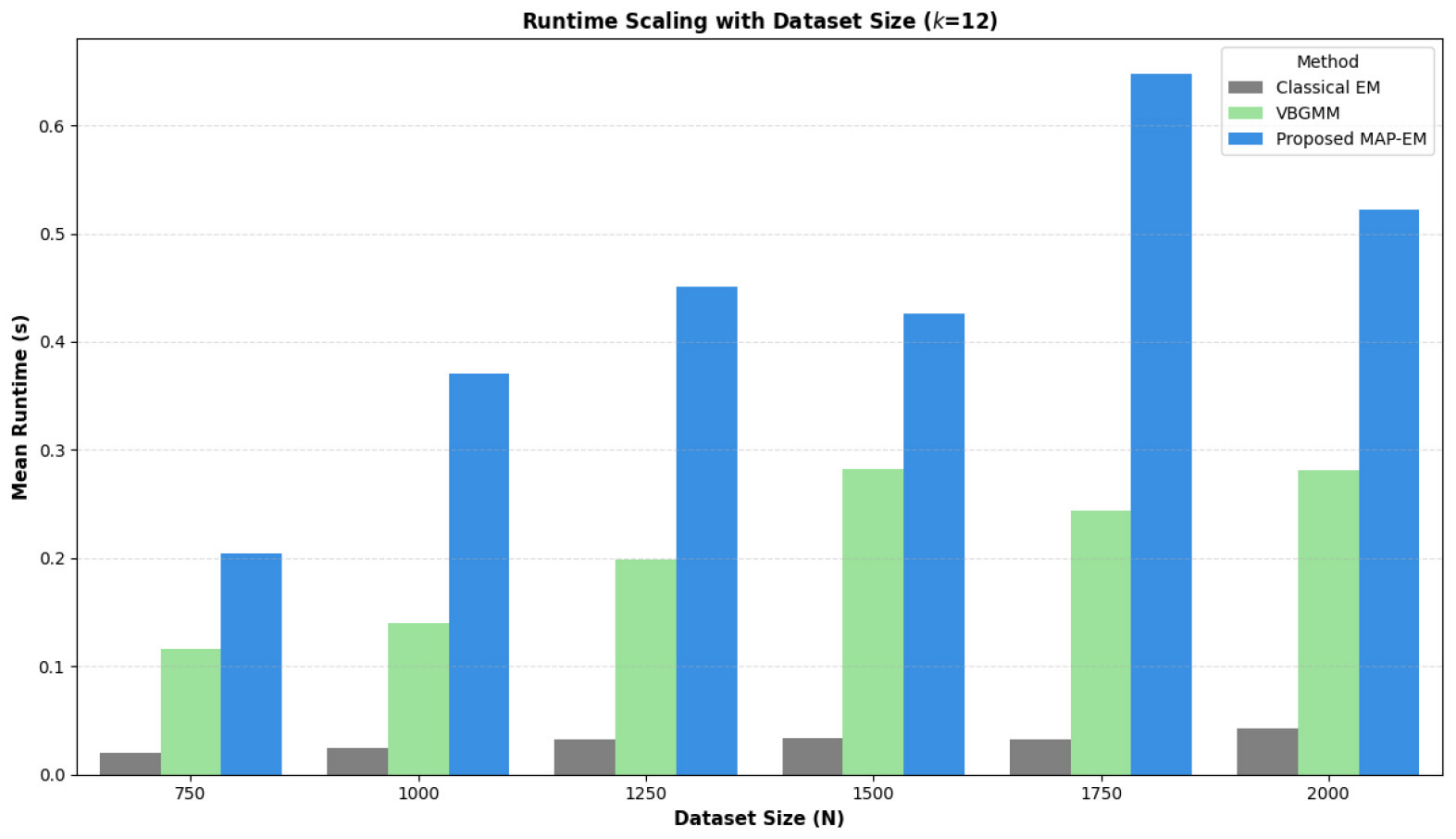
Runtime scaling of EM–GMM, VB–GMM, and the proposed MAP–EM model with increasing dataset size *N*, while the number of clusters is fixed at *K* = 12.

Table 10 and Figure 8 show the effect of increasing dataset size while fixing the number of clusters at *K* = 12. Runtime generally increased with *N* for all three methods. The increase in runtime was not perfectly monotonic. EM–GMM remained the fastest method throughout, increasing only from 0.0100 s at *N* = 100 to 0.0236 s at *N* = 2000. The proposed MAP–EM method required higher runtime, ranging from 0.1120 s to 0.5020 s, while VB–GMM showed intermediate runtime, increasing from 0.0207 s to 0.1811 s.

**Table 10:** Mean runtime (seconds) of EM–GMM, VB–GMM, and the proposed MAP–EM model for the scalability experiment with varying dataset size at fixed *K* = 12.

Dataset size (*N*)	EM–GMM	VB–GMM	Proposed MAP–EM
100	0.0100	0.0207	0.1120
250	0.0143	0.0891	0.3453
500	0.0138	0.0835	0.3013
750	0.0147	0.0848	0.1403
1,000	0.0184	0.1022	0.2528
1,250	0.0177	0.1346	0.2976
1,500	0.0244	0.2097	0.5020
1,750	0.0236	0.1823	0.4020
2,000	0.0236	0.1811	0.3175

**Figure 9:**
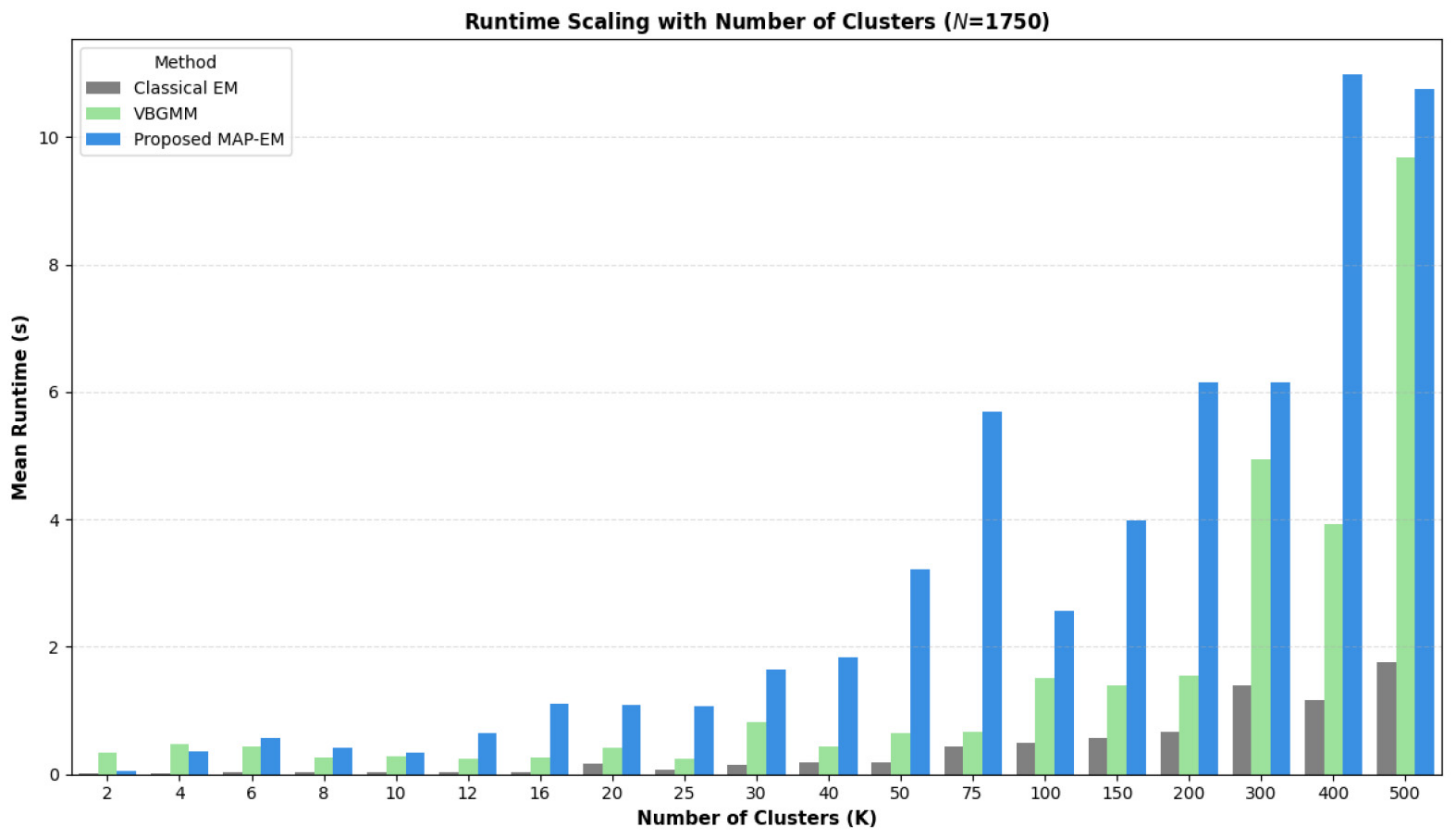
Runtime scaling of EM–GMM, VB–GMM, and the proposed MAP–EM model with increasing number of clusters *K*, while the dataset size is fixed at *N* = 1750.

Table 11 and Figure 9 show the effect of increasing the number of clusters while fixing the dataset size at *N* = 1750. Runtime generally increased as *K* grew for all methods. EM–GMM again showed the lowest computational cost, increasing from 0.0079 s at *K* = 2 to 0.8027 s at *K* = 500. The proposed MAP–EM model exhibited the highest runtime, increasing from 0.0331 s at *K* = 2 to 6.1223 s at *K* = 500, while VB–GMM increased from 0.2201 s to 5.9259 s over the same range. These results indicate that the computational burden increases with model complexity across all three methods, with the proposed MAP–EM incurring additional runtime due to its richer modeling structure.

**Table 11:** Mean runtime (seconds) of EM–GMM, VB–GMM, and the proposed MAP–EM model in the dataset-size scalability experiment, where the sample size *N* varies, and the number of clusters is fixed at *K* = 12.

#Clusters (*K*)	EM–GMM	VB–GMM	Proposed MAP–EM
2	0.007888	0.220084	0.033146
4	0.010589	0.235463	0.123731
6	0.049220	0.358417	0.348422
8	0.015599	0.170725	0.262630
10	0.026759	0.185646	0.232018
12	0.023551	0.182251	0.401991
16	0.027054	0.167768	0.477522
20	0.043398	0.161597	0.600291
25	0.055231	0.188350	1.103649
30	0.081081	0.501090	0.824123
40	0.198296	0.257432	1.617653
50	0.168383	0.477930	1.530247
75	0.211155	0.553353	3.071527
100	0.330742	0.949910	1.667950
150	0.318998	0.936788	2.625579
200	0.663936	1.140700	3.321308
300	0.644461	2.729075	4.197978
400	0.960207	2.790089	6.006199
500	0.802735	5.925882	6.122277

In terms of iteration count, EM–GMM converged in 2 iterations for most of the reported settings, although a few cases required slightly more iterations, such as *N* = 1500 with *K* = 12 and some larger *K* settings at *N* = 1750. Conversely, VB–GMM and the proposed MAP–EM model typically required more iterations than EM–GMM, particularly in the larger-*K* settings, owing to their more involved update mechanisms.

In terms of memory usage, the proposed MAP–EM model stores the usual mixture parameters together with the graph Laplacian associated with the regularization term. Relative to standard EM, the main additional memory cost arises from the graph representation. When the Laplacian is stored in sparse form, the memory requirement scales primarily with the number of non-zero edges, rather than with the number of non-zero entries in a dense matrix representation, making the method more practical for large sparse graphs. Thus, although the proposed MAP–EM is more memory-intensive than EM–GMM, sparse storage keeps the additional memory demand manageable in large sparse-graph settings.

The proposed MAP–EM model is computationally more expensive than standard *k*–means and conventional EM-based clustering because it includes posterior parameter estimation, feature-graph regularization, noise modeling, and split–merge adaptation. Let *n* denote the number of documents, *d* the feature dimension, *K* the number of clusters, and *T* the number of EM iterations. In a reduced or diagonal-covariance implementation, the main EM update has an approximate cost of *O*(*TnKd*). With full covariance estimation, the cost increases because computing covariances and performing matrix operations become more expensive in high-dimensional spaces. The graph-regularized mean update incurs additional cost proportional to the number of non-zero edges in the feature graph, denoted by |*E*|. Therefore, the runtime increases with the number of documents, vocabulary size, clusters, graph density, and split–merge refinement moves.

The scalability analysis in this study was conducted on synthetic datasets to examine these effects under controlled conditions. The results show that MAP–EM remains feasible for small- to medium-sized datasets, but it is slower than simpler baselines due to the additional regularization and model-adaptation steps. In the present implementation, the method is most practical when the dataset size and feature dimension are moderate, and when stability, noise handling, and adaptive cluster-number estimation are important enough to justify the extra computational cost.

To improve scalability in larger document clustering tasks, several optimization strategies can be used. Sparse TF–IDF document-term matrices and vocabulary pruning can reduce memory usage and computation during feature representation and graph construction. The feature graph can be constructed using sparse *k*-nearest-neighbor connections rather than a dense graph, and approximate nearest-neighbor search can reduce graph construction time for large vocabularies. During MAP–EM updates, diagonal or tied covariance approximations can reduce the cost of covariance estimation and help retain some of the computational benefit of sparse text representations, since full covariance estimation can lead to dense matrix operations in high-dimensional spaces. Mini-batch or warm-started EM updates may also be used when the number of documents is large. Furthermore, split–merge operations can be applied only after selected EM iterations or restricted to the most uncertain components. These strategies can make the proposed MAP–EM model more practical for larger document collections.

### 6.5 Experiment on benchmark dataset

The three text-based corpora—Reuters–R8 (Lewis, 1997), BBC sports, and BBC dataset—were used to demonstrate the model's performance in sparse high-dimensional spaces. The BBC and BBC Sports datasets were obtained from the Machine Learning Group at University College Dublin repository (Greene and Cunningham, 2006). Textual datasets were pre-processed using a standard pipeline consisting of tokenization, lower-casing, punctuation removal, stop-word removal, and lemmatization. The processed documents were converted into a TF–IDF-weighted document-term matrix and normalized to unit length before clustering.

#### 6.5.1 Hyperparameter selection

To improve transparency and reproducibility, the hyperparameters of the proposed MAP–EM model were examined using a predefined coarse grid search on the Reuters–R8 dataset. Reuters–R8 was used as the development dataset because it is the largest benchmark corpus considered in this study and contains a broader document-clustering structure.

The grid search was carried out over the proposed MAP–EM parameters: the graph regularization weight γ, the bounded uniform-noise weight β_noise_, the split–merge complexity penalty β, the feature-graph neighborhood size *k*_NN_, and the maximum number of split–merge refinement moves. The search space contained 243 configurations, as summarized in Table 12.

**Table 12:** Hyperparameter search space used for the proposed MAP–EM model.

Hyperparameter	Search space	Role in the model
γ	0.001, 0.01, 0.1	Controls the strength of the feature-graph Laplacian regularization.
β_noise_	0.0, 0.05, 0.1	Controls the contribution of the bounded uniform noise component.
β	0.5, 1.0, 2.0	Controls the BIC-type complexity penalty in the split–merge criterion.
*kNN*	5, 10, 15	Determines the sparsity of the feature similarity graph.
Maximum split–merge moves	0, 3, 6	Limits the number of adaptive refinement moves in each run.

The grid-search results show that the highest ARI, NMI, and accuracy were obtained with γ = 0.001, β_noise_ = 0.10, β = 0.5, *k*_NN_ = 5, and maximum split–merge moves equal to 3. This setting achieved ARI = 0.7136, NMI = 0.6196, accuracy = 0.8065, and recovered 7 clusters, giving a cluster-count error of 1. A second strong configuration, with γ = 0.10, β_noise_ = 0.00, β = 0.5, *k*_NN_ = 15, and maximum split–merge moves equal to 6, achieved ARI = 0.6892, NMI = 0.5752, accuracy = 0.7940, and recovered the correct number of clusters. These representative configurations are shown in Table 13.

**Table 13:** Representative configurations from the Reuters–R8 grid search.

Configuration	γ	β_noise_	β	*kNN*	Moves	ARI	NMI	*K*_error_
Highest ARI/NMI/accuracy	0.001	0.10	0.5	5	3	0.7136	0.6196	1
Exact cluster-count recovery	0.10	0.00	0.5	15	6	0.6892	0.5752	0
Stable transferable setting	0.01	0.05	1.0	5	6	0.3194	0.2902	0

The final parameter choice was not based only on maximizing a single external score. Instead, the grid search was interpreted together with the sensitivity analysis to identify settings that balance clustering quality, cluster-count recovery, and numerical stability. This distinction is important because the best ARI configuration did not recover the exact number of clusters, whereas some configurations that did recover the exact cluster count used stronger graph construction or disabled the noise component. Therefore, the selected setting was treated as a practical transferable configuration rather than a universally optimal parameter choice.

The same selected MAP–EM configuration was then transferred to BBC and BBC sports without dataset-specific retuning. This was done to reduce dataset-specific overfitting and to make the benchmark comparison more transparent. Thus, the reported benchmark results reflect the behavior of one fixed MAP–EM setting across all three corpora, rather than separately tuned settings for each dataset.

#### 6.5.2 Results on benchmark dataset

In the reported experiment, no dimensionality reduction technique was applied, such as PCA, SVD, or LSA. Instead, for each dataset, a fixed number of terms was retained after preprocessing and vocabulary filtering, as reported in Table 14. Thus, all methods were evaluated on the same TF–IDF representation to ensure a fair comparison. Table 14 shows the differences between the three datasets in terms of scale and vocabulary richness. The baseline algorithms include *k*-means, spectral clustering, expectation-maximization Gaussian mixture model (EM–GMM), variational Bayesian Gaussian mixture model (VB–GMM), and Laplacian regularized Gaussian mixture model (LapGMM). The clustering performance was assessed using the adjusted Rand index (ARI), normalized mutual information (NMI), F1 score, accuracy, purity, silhouette score, and Dunn index.

**Table 14:** Characteristics of the text datasets used for document clustering experiment, including dataset size, number of classes, and number of terms used.

Dataset	Documents	#Clusters	#Terms
Reuters–R8	7,674	8	40,000
BBC sports	737	5	10,000
BBC News	2,225	5	20,000

For the baseline methods, all algorithms were evaluated using the same preprocessed TF–IDF document-term matrices. The number of clusters was fixed to the known number of ground-truth categories for each benchmark dataset. K–Means and EM–GMM were run with multiple random initializations, and the best solution according to their respective objective functions was retained. Spectral Clustering, VB–GMM, and LapGMM were evaluated under the same feature representation and category-count setting. Thus, the comparison was kept consistent across methods. At the same time, the proposed MAP–EM model has more tunable components than simpler baselines, and this additional tuning burden is acknowledged as a practical limitation.

In the benchmark experiments, the sensitivity to hyperparameter choices was mainly observed through the balance between clustering quality and cluster-count recovery. On Reuters–R8, the best external-score setting achieved ARI = 0.7136, NMI = 0.6196, and accuracy = 0.8065, but it recovered seven clusters instead of the true 8 classes. Conversely, another configuration recovered the correct number of clusters and still produced competitive scores, with ARI = 0.6892, NMI = 0.5752, and accuracy = 0.7940. This shows that the hyperparameters not only affect clustering agreement but also influence the estimated model order.

The graph regularization weight γ, the noise weight β_noise_, and the split–merge settings had the clearest practical effect. Smaller γ values gave stronger external clustering scores on Reuters–R8, while stronger graph construction helped recover the correct number of clusters in some settings. Similarly, the noise component improved robustness in some configurations, but larger noise weights could absorb borderline documents, thereby affecting cluster recovery. For this reason, the final setting was selected as a conservative transferable configuration rather than the single highest-scoring configuration. This choice is also important for BBC and BBC sports, where the corpora are smaller and more sensitive to over-smoothing, noise weighting, and split–merge refinement.

The benchmark experiments were conducted on BBC, BBC sports, and Reuters–R8 to examine how MAP–EM performs on sparse, high-dimensional document representations. The proposed method was compared with K–Means, Spectral Clustering, EM–GMM, VB–GMM, and LapGMM using ARI, NMI, F1, accuracy, and Purity. These baseline methods were selected to represent distance-based clustering, graph-based clustering, classical mixture modeling, Bayesian mixture modeling, and Laplacian-regularized mixture modeling.

**Figure 10:**
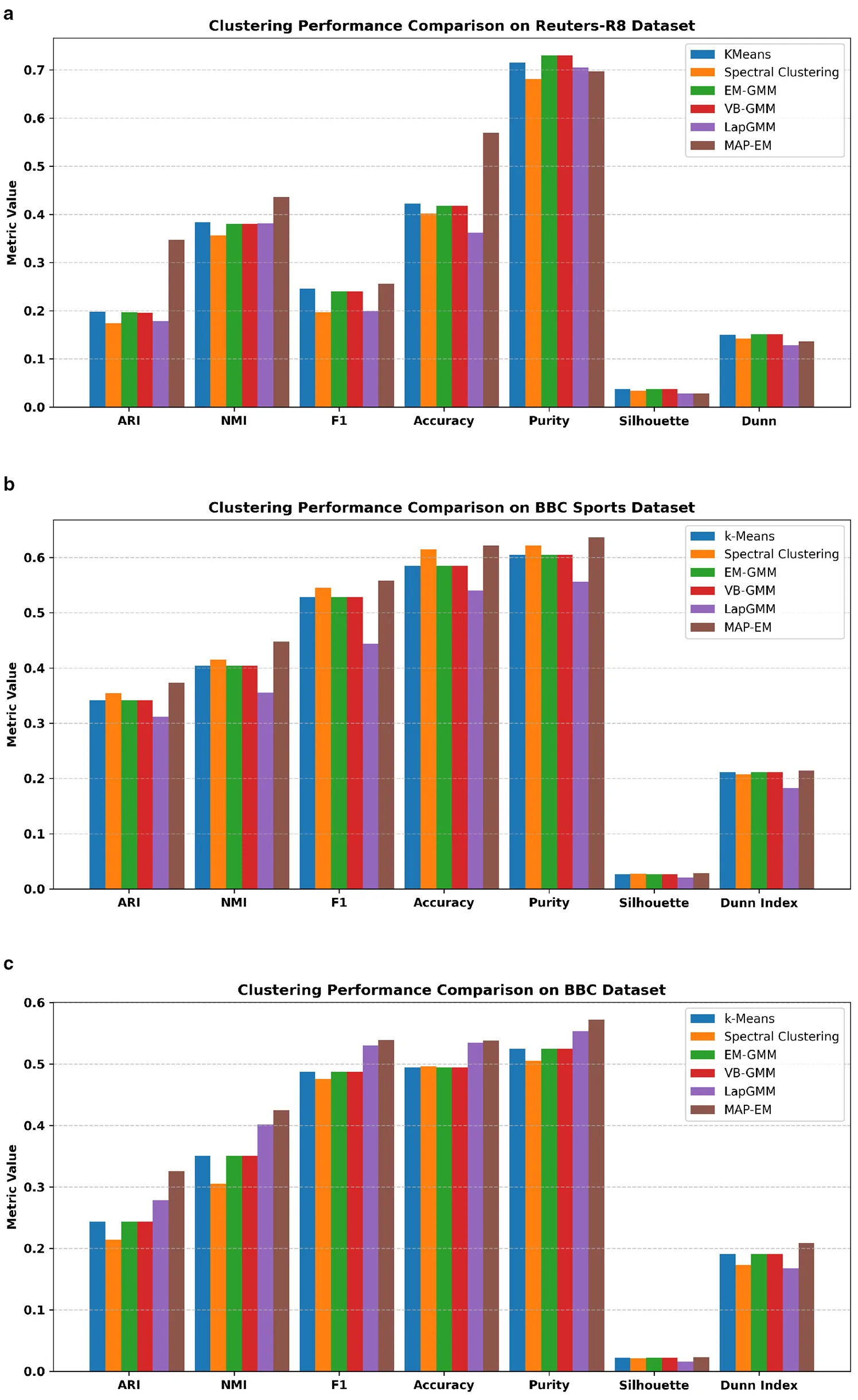
Comparison of clustering performance across the Reuters–R8, BBC sports, and BBC text datasets using ARI, NMI, F1 score, clustering accuracy, purity, Silhouette score, and Dunn index. **(a)** Reuters–R8 dataset. **(b)** BBC sports dataset. **(c)** BBC dataset.

Tables 15–17 summarize the performance of the proposed MAP–EM model, *k*-means, spectral clustering, EM–GMM, VB–GMM, and LapGMM on the three text datasets using ARI, NMI, F1 score, clustering accuracy, purity, Silhouette score, and Dunn index as evaluation measures. Figure 10 provides a visual comparison of the competing methods across all evaluation metrics and datasets, illustrating that their relative performance varies with both the dataset and the metric considered. The results show that the relative performance of the competing methods is both dataset-dependent and metric-dependent.

**Table 15:** Performance of the competing clustering algorithms on the Reuters–R8 dataset using ARI, NMI, F1 score, clustering accuracy, purity, Silhouette score, and Dunn index.

Method	ARI	NMI	F1	Accuracy	Purity	Silhouette	Dunn
*k*–Means	0.1620	0.3782	0.1871	0.3322	0.6985	**0.0372**	0.1467
Spectral Clustering	0.1729	0.3602	0.1962	0.4013	0.6810	0.0335	0.1411
EM–GMM	0.1054	0.3213	0.1479	0.3019	0.7284	0.0371	0.1561
VB–GMM	0.3171	**0.4452**	**0.2569**	0.5007	**0.7685**	0.0364	**0.1564**
LapGMM	0.1783	0.3807	0.1989	0.3620	0.7048	0.0278	0.1280
**MAP–EM (proposed)**	**0.3470**	0.4354	0.2552	**0.5693**	0.6968	0.0285	0.1367

Bold values indicate the best performing result for each comparison.

**Table 16:** Performance of the competing clustering algorithms on the BBC sports dataset using ARI, NMI, F1 score, clustering accuracy, purity, Silhouette score, and Dunn index.

Method	ARI	NMI	F1	Accuracy	Purity	Silhouette	Dunn
*k*-Means	**0.3788**	**0.4551**	0.5283	0.5744	0.6323	**0.0291**	**0.2239**
Spectral Clustering	0.3567	0.4168	0.5460	0.6160	0.6227	0.0274	0.2073
EM–GMM	0.3072	0.4215	**0.5624**	0.5549	0.6350	0.0244	0.1829
VB–GMM	0.2768	0.2888	0.3970	0.4952	0.5210	0.0257	0.2115
LapGMM	0.3120	0.3550	0.4433	0.5400	0.5563	0.0204	0.1824
**MAP–EM (proposed)**	0.3731	0.4479	0.5579	**0.6214**	**0.6364**	0.0282	0.2138

Bold values indicate the best performing result for each comparison.

**Table 17:** Performance of the competing clustering algorithms on the BBC dataset using ARI, NMI, F1 score, clustering accuracy, purity, Silhouette score, and Dunn index.

**Method**	ARI	NMI	F1	Accuracy	Purity	Silhouette	Dunn
*k*-Means	0.2579	0.3759	0.5279	0.5361	0.5478	0.0219	0.1852
Spectral Clustering	0.2137	0.3049	0.4755	0.4957	0.5047	0.0213	0.1730
EM–GMM	0.2450	0.3368	0.4921	0.5101	0.5195	0.0224	0.1777
VB–GMM	0.2037	0.3174	0.4824	0.4939	0.5096	0.0213	0.1807
LapGMM	0.2780	0.4014	0.5303	0.5344	0.5533	0.0157	0.1675
**MAP–EM (proposed)**	**0.3256**	**0.4248**	**0.5390**	**0.5384**	**0.5721**	**0.0230**	**0.2085**

Bold values indicate the best performing result for each comparison.

On Reuters–R8, the proposed MAP–EM model achieved the highest ARI (0.3470) and accuracy (0.5693), outperforming the second-best methods by approximately 5.8% and 2.8%, respectively. However, VB–GMM obtained the highest NMI (0.4452), F1 score (0.2569), and Dunn index (0.1564), while LapGMM achieved the highest Purity (0.7048) and K–Means produced the highest Silhouette score (0.0372). These results suggest that MAP–EM yields the strongest recovery of the underlying class structure, as measured by ARI and accuracy, whereas VB–GMM produces clusters with slightly stronger information-theoretic agreement and internal compactness. The fact that the best scores are distributed across several methods indicates that Reuters–R8 remains a challenging dataset with overlapping topic boundaries and heterogeneous cluster densities. Nevertheless, MAP–EM consistently ranks among the strongest methods on the principal external validation measures, demonstrating that the combination of MAP regularization and graph-based smoothing improves robustness on large-scale sparse text collections.

On BBC sports, no single method dominated all metrics. *K*-means achieved the highest ARI (0.3788), NMI (0.4551), Silhouette score (0.0291), and Dunn index (0.2239), while MAP–EM obtained the highest accuracy (0.6214) and Purity (0.6364). EM–GMM achieved the highest F1 score (0.5624). Compared with K–Means, MAP–EM improved accuracy by approximately 4.4% and Purity by about 2.0%, but its ARI and NMI were lower. This pattern suggests that BBC sports contains categories with substantial vocabulary overlap, making cluster boundaries less distinct in TF–IDF space. Under such conditions, methods optimized for centroid separation can achieve higher geometric clustering scores, whereas MAP–EM yields more reliable document assignments, as reflected in accuracy and Purity. The results therefore indicate that the advantage of MAP–EM on BBC sports lies in assignment quality rather than in uniform superiority across all clustering criteria.

On the BBC dataset, MAP–EM achieved the strongest overall performance among all competing methods, obtaining the highest ARI (0.3256), NMI (0.4248), F1 score (0.5390), accuracy (0.5384), Purity (0.5721), Silhouette score (0.0230), and Dunn index (0.2085). Relative to the second-best method, the gains were approximately 7.8% in ARI, 4.8% in NMI, 2.7% in F1 score, 2.8% in accuracy, and 2.0% in Purity. LapGMM was generally the closest competitor but remained below MAP–EM on every reported metric. Unlike Reuters–R8 and BBC sports, where leadership was shared among several methods, BBC shows a consistent advantage for MAP–EM across both external and internal validation measures. This suggests that the MAP prior and feature-graph Laplacian effectively guide the EM optimization process toward more stable local optima, reducing overconfident assignments while preserving cluster separability.

The results reveal three distinct behaviors. First, MAP–EM is the strongest method on BBC, consistently outperforming all competitors across all metrics. Second, on Reuters–R8 it achieves the best external clustering agreement according to ARI and accuracy while remaining competitive on the remaining measures. Third, on BBC sports, its performance is mixed, with different methods leading on different metrics. These findings indicate that the proposed regularization strategy is particularly beneficial when document clusters exhibit moderate topic overlap and sparse high-dimensional representations, while its advantages become less pronounced when categories are highly similar and cluster boundaries are weakly separated.

Although this study uses sparse TF–IDF document representations, the MAP–EM framework is not limited to this feature space. In principle, it can be extended to other text representations, such as Doc2Vec embeddings, aggregated Word2Vec or fastText vectors, and contextual dense embeddings from transformer-based models such as sentence-BERT. In these settings, the main components of the method, namely the NIW prior, uniform noise component, and split–merge refinement, would remain relevant. However, their practical behavior may differ from that observed with TF–IDF because dense embeddings are usually lower-dimensional and more semantically structured. This may influence covariance estimation, graph Laplacian construction, and cluster separation. In this regard, sparse neural text representations may offer a useful middle ground, combining some of the structural advantages of sparsity with richer semantic information. A systematic empirical comparison across these alternative representations would therefore be a valuable direction for future research.

### 6.6 Statistical significance analysis

To determine whether the observed performance differences were statistically significant, pairwise significance tests were conducted between MAP–EM and each baseline method across repeated runs. Since the results were paired by run index and normality could not be assumed for all metrics, the Wilcoxon signed-rank test was used as a non-parametric paired test. Bonferroni correction was applied at α = 0.05 to control for multiple comparisons. The significance level was set at α = 0.05, a commonly used threshold for determining statistical significance in experimental studies. Since MAP–EM was compared with multiple baseline methods across several datasets and evaluation metrics, a correction for multiple testing was required to reduce the probability of false-positive conclusions. Therefore, the Bonferroni correction was applied to the Wilcoxon signed-rank test results. This correction is conservative, but it provides a stricter and more reliable basis for interpreting the statistical evidence across the 75 method-level comparisons.

#### 6.6.1 Wilcoxon signed-rank test with Bonferroni correction

Table 18 summarizes the statistical comparison across the BBC, BBC sports, and Reuters–R8 datasets. The analysis includes 75 method-level comparisons, obtained from three datasets, five evaluation metrics, and five baseline methods. These comparisons produced 1,115 valid paired run-wise observations after matching MAP–EM and each baseline by run index and removing unavailable entries. The full design allows up to 1,125 paired observations; however, 10 entries were unavailable after run-wise matching.

**Table 18:** Statistical significance analysis for MAP–EM against baseline methods.

**Criterion**	Count	Percentage
Method-level comparisons	75	100.0%
Significant positive differences	45	60.0%
Significant negative differences	1	1.3%
Non-significant comparisons	29	38.7%
Valid paired run comparisons	1,115	100.0%
MAP–EM wins	891	79.9%
MAP–EM losses	206	18.5%

As shown in Table 18, MAP–EM achieved statistically significant positive differences in 45 out of 75 method-level comparisons. One comparison showed a significant negative difference, while 29 comparisons were not statistically significant after Bonferroni correction. At the run level, MAP–EM obtained higher scores in 891 out of 1,115 valid paired comparisons, corresponding to a win rate of 79.9%.

The dataset-wise significance results are summarized in Table 19. The results show that the statistical support for MAP–EM varies across datasets. Reuters–R8 gives the strongest evidence, with 22 significant positive comparisons out of 25. The BBC dataset also supports the proposed method, with 15 significant positive comparisons and no significant negative comparison. In BBC Sports, the results are more mixed, with eight significant positive comparisons, one significant negative comparison, and 16 non-significant comparisons.

**Table 19:** Dataset-wise summary of statistical significance results.

Dataset	Total comparisons	Significant positive	Significant negative	Non-significant
BBC	25	15	0	10
BBC sports	25	8	1	16
Reuters–R8	25	22	0	3

This pattern supports the interpretation of the experiments. MAP–EM shows statistically significant advantages in several benchmark settings, but the improvements are not consistent across all datasets and metrics. The weaker significance pattern on BBC sports may be partly due to the size and structure of the dataset. BBC sports is smaller than the other benchmark datasets, and its categories share several common vocabulary terms. For example, words such as team, match, player, season, and win may appear across different sports categories, reducing cluster separation in the TF–IDF feature space. As a result, the performance gap between MAP–EM and the baseline methods narrows on some metrics. Therefore, these results should not be interpreted as a failure of the proposed method. Rather, they show that the advantage of MAP–EM depends on the dataset structure and the separability of the underlying topics.

To complement the pairwise significance analysis, summary statistics were computed for MAP–EM across repeated runs. Table 20 reports the mean, standard deviation, and 95% confidence interval over 15 runs on the BBC, BBC sports, and Reuters–R8 datasets.

**Table 20:** Summary statistics of MAP–EM performance across 15 repeated runs.

Dataset	Metric	Mean	SD	95% CI
BBC	ARI	0.2978	0.0307	[0.2808, 0.3148]
BBC	NMI	0.3992	0.0346	[0.3800, 0.4184]
BBC	F1	0.5300	0.0158	[0.5213, 0.5387]
BBC	Accuracy	0.5345	0.0104	[0.5287, 0.5403]
BBC	Purity	0.5595	0.0189	[0.5490, 0.5700]
BBC sports	ARI	0.3780	0.0065	[0.3744, 0.3816]
BBC sports	NMI	0.4513	0.0073	[0.4473, 0.4553]
BBC sports	F1	0.5676	0.0053	[0.5647, 0.5705]
BBC sports	Accuracy	0.6071	0.0114	[0.6008, 0.6134]
BBC sports	Purity	0.6325	0.0050	[0.6297, 0.6353]
Reuters–R8	ARI	0.4412	0.0478	[0.4147, 0.4677]
Reuters–R8	NMI	0.4863	0.0411	[0.4635, 0.5091]
Reuters–R8	F1	0.3090	0.0563	[0.2778, 0.3402]
Reuters–R8	Accuracy	0.6132	0.0402	[0.5909, 0.6355]
Reuters–R8	Purity	0.7698	0.0370	[0.7493, 0.7903]

The results show that MAP–EM is relatively stable on BBC sports, with small standard deviations across all five metrics. For example, ARI is (0.3780 ± 0.0065), and NMI is (0.4513 ± 0.0073). The BBC dataset also shows moderate stability, with mean values of (0.2978) for ARI, (0.3992) for NMI, and (0.5300) for F1. On Reuters–R8, MAP–EM achieves a stronger overall performance in ARI, NMI, accuracy, and purity, although the standard deviations are slightly larger than those observed for BBC sports.

The confidence intervals describe the run-to-run variation in MAP–EM performance. The Wilcoxon signed-rank test evaluates whether the observed differences between MAP–EM and the baseline methods are statistically significant. Taken together, these findings support a balanced interpretation. MAP–EM is statistically supported in several settings, but its superiority is dataset-dependent and metric-dependent rather than universal.

##### 6.6.1.1 Robustness to multiple-testing correction

To assess the robustness of the statistical conclusions, the pairwise Wilcoxon tests were corrected using both the Bonferroni procedure and the Benjamini–Hochberg false discovery rate (FDR) method. Under the Bonferroni correction, 46 pairwise comparisons remained statistically significant, whereas the FDR procedure identified 47 significant comparisons. No statistically significant negative comparisons were observed under FDR control. The close agreement between these two correction procedures indicates that the statistical superiority of the proposed MAP–EM framework is not sensitive to the choice of multiple-testing correction.

#### 6.6.2 Effect size analysis (Cohen's *d*_*z*_)

Statistical significance alone does not quantify the practical magnitude of the observed improvements. Therefore, paired effect sizes were computed using Cohen's *d*_*z*_ for all statistically significant positive comparisons. Following Cohen (1988), values of approximately 0.2, 0.5, and 0.8 correspond to small, medium, and large effects, respectively. Across the statistically significant comparisons, the proposed MAP–EM framework achieved a median paired effect size of *d*_*z*_ = 1.54 (interquartile range: 1.16–2.40), indicating a consistently large practical improvement over the competing clustering methods. These results complement the Wilcoxon significance tests by demonstrating that the observed improvements are not only statistically significant but also practically meaningful.

#### 6.6.3 Bootstrap confidence intervals for win rates

To further assess the stability of the observed performance, percentile bootstrap confidence intervals were computed from 100,000 resamples of the paired-comparison outcomes. Ties were retained in the denominator and treated as non-wins, providing a conservative estimate of the win rate. As summarized in Table 21, the proposed MAP–EM framework consistently achieved high win rates across all benchmark datasets. Reuters–R8 exhibited the highest win rate (92.53%; 95% CI: 89.87%–95.20%), followed by BBC (84.66%; 95% CI: 80.82%–88.22%). Although the improvement on BBC Sports was comparatively smaller, the win rate remained above 60% (62.67%; 95% CI: 57.87%–67.47%). Across all 1,115 paired comparisons, the overall win rate was 79.91%, with a bootstrap 95% confidence interval of 77.49%–82.24%. The relatively narrow confidence intervals indicate that the observed win rates are stable across repeated resampling, providing additional evidence for the robustness of the proposed MAP–EM framework.

**Table 21:** Bootstrap 95% confidence intervals for MAP–EM win rates across paired comparisons.

Dataset	Comparisons	Wins (%)	95% CI
BBC	365	84.66	80.82–88.22
BBC Sports	375	62.67	57.87–67.47
Reuters–R8	375	92.53	89.87–95.20
Total	1,115	79.91	77.49–82.24

## 7 Conclusion

Classical GMM-EM remains a powerful and interpretable clustering method, but it is sensitive to initialization, prone to likelihood degeneracy, ineffective at exploiting feature structure, and inflexible in determining the number of clusters. The proposed MAP–EM model addresses these limitations by incorporating normal–inverse–Wishart priors, graph-based regularization, a noise-handling component, and an adaptive model-selection heuristic within a unified EM framework that preserves monotone ascent while alleviating several well-known weaknesses of classical Gaussian mixtures.

Empirical results on both synthetic and benchmark datasets indicate that MAP–EM achieves stable convergence and competitive clustering performance. However, its effectiveness depends on dataset structure and the interaction among its components. The synthetic-data experiments show that some configurations remain robust to overlap and outliers, whereas non-spherical settings continue to pose challenges. The broader experimental analysis, including sensitivity, scalability, and runtime analysis, further clarifies the practical behavior of the model. On benchmark text datasets, MAP–EM delivered strong results, outperforming all competing methods across all reported metrics on the BBC dataset, while showing more metric-dependent performance on Reuters-R8 and BBC sports. Repeated run analysis and formal statistical tests further support its stability and competitiveness.

The study also identified practical limitations. The method is sensitive to hyperparameter selection, particularly the graph regularization weight, noise contribution, and split-merge penalty, and its effectiveness depends on the quality and connectivity of the feature graph. In highly sparse, poorly connected, strongly non-Gaussian, or heavily imbalanced settings, additional preprocessing, dimensionality reduction, graph reconstruction, or alternative feature representations may be necessary. Moreover, MAP–EM is computationally more demanding than standard k-means and conventional EM-based clustering due to the added costs of regularization, noise modeling, and split-merge refinement.

Future work may further improve the proposed MAP–EM framework in several ways. One promising direction is the development of adaptive hyperparameter selection strategies that automatically adjust the graph regularization weight, noise contribution, and split–merge penalty during optimization. The quality of the feature graph could also be enhanced by incorporating approximate nearest-neighbor methods, semantic embeddings, or hybrid lexical–semantic graph constructions to better capture relationships among terms. Another direction is to relax the Gaussian mixture assumption by exploring heavy-tailed distributions, non-Gaussian mixture models, or neural mixture models that can better represent complex document structures. From a computational perspective, scalability could be improved through mini-batch MAP–EM updates, sparse matrix implementations, parallel E-step computation, and more efficient split–merge strategies. Finally, evaluating the proposed framework on larger and more diverse document collections would provide further insight into its generalization and help identify scenarios in which the additional model complexity offers meaningful advantages over simpler clustering approaches.

## Data Availability

Publicly available datasets were analyzed in this study. This data can be found here: https://kdd.ics.uci.edu/databases/reuters21578/reuters21578.html and http://mlg.ucd.ie/datasets/bbc.html.
